# Strain-resolved metabolomic chemotyping identifies tryptophan and γ-glutamyl peptides as MGO-AGEs breaking postbiotic compounds for carbonyl stress mitigation

**DOI:** 10.1080/19490976.2026.2728833

**Published:** 2026-09-12

**Authors:** Su-Hyun Kim, Seong-Min Hong, Eun Yoo Lee, Seul-Ah Kim, Nam Soo Han, Sun Yeou Kim, Choong Hwan Lee

**Affiliations:** a Department of Bioscience and Biotechnology, Konkuk University, Seoul, Republic of Korea; b Division of Food Science and Technology and Institute of Agriculture and Life Science, Gyeongsang National University, Jinju, Gyeongnam, Republic of Korea; c College of Pharmacy and Gachon Institute of Pharmaceutical Science, Gachon University, Incheon, Republic of Korea; d Brain Korea 21 Center for Bio-Health Industry, Division of Animal, Horticultural, and Food Sciences, Chungbuk National University, Cheongju, Republic of Korea; e Research Institute for Bioactive-Metabolome Network, Konkuk University, Seoul, Republic of Korea

**Keywords:** Activity metabolomics, metabolomics-based chemotyping, postbiotic extract, microbial metabolite, MGO-AGEs breaking activity, carbonyl stress, depressive-like behaviors, gut-brain axis, intestinal metabolome, *γ*-glutamyl peptides

## Abstract

Carbonyl stress, characterized by pathological accumulation of reactive dicarbonyl species such as methylglyoxal (MGO), disrupts mitochondrial function and drives formation of advanced glycation end-products (AGEs), and is implicated in chronic neurobehavioral and metabolic disorders. Here, we established a strain-resolved activity metabolomics framework to rationally select postbiotic extracts with MGO-AGEs breaking activity. We screened a postbiotic library of 177 intracellular metabolite extracts from 113 bacterial strains by untargeted metabolite profiling and *in vitro* MGO-AGEs breaking activity. Comparative analysis revealed that strain-level chemotypes within *Limosilactobacillus fermentum* showed marked differences in functional activity that were not apparent at the species level. Using this framework, we selected the postbiotic extract LFEAN031 based on strain-level enrichment of MGO-AGEs breaking compounds, including tryptophan, asparagine, and three *γ*-glutamyl peptides (GGPs), and evaluated its efficacy in an MGO-induced carbonyl stress mouse model. Oral LFEAN031 administration restored MGO-induced colon length shortening and increased colonic expression of the tight junction protein ZO-1, while lowering serum interleukin-6 (IL-6), cortisol, and reducing hypothalamic glucocorticoid receptor expression, indicating attenuation of hypothalamic-pituitary-adrenal (HPA) axis hyperactivation and inflammatory responses. Depressive- and anxiety-like behaviors also improved. These phenotypic improvements were accompanied by altered cecal GGP-related pathways, with increased levels of the three bioactive GGPs and redox-related metabolites, alongside a shift in tryptophan metabolism toward protective indole derivatives and 5-hydroxytryptophan, with reduced levels of the neurotoxic metabolites 3-indoxyl sulfate and xanthurenic acid. These findings provide proof-of-concept for a metabolite-guided strategy for postbiotic discovery and demonstrate the capacity of microbial metabolites to modulate gut-brain axis function under carbonyl stress.

## Graphical abstract

**Figure:**
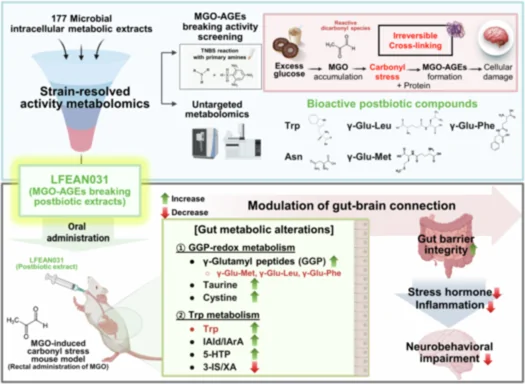

## Introduction

The gut microbiota plays a crucial role in regulating host physiology through bidirectional communication along the gut–brain axis.^1^ Increasing evidence indicates that microbial dysbiosis contributes to a wide range of disorders including gastrointestinal dysfunction, neuropsychiatric disorders, and neurodegenerative diseases.^2^ ^,^ ^3^ Specifically, *Limosilactobacillus fermentum* and other lactic acid bacteria have been reported to be depleted in patients with major depressive disorders and disorders of gut-brain interaction.^1-3^ As candidate modulators of these neuropsychiatric conditions, diverse microorganisms, such as *Bifidobacterium longum*, *Lacticaseibacillus rhamnosus,* and *Limosilactobacillus* *reuteri*, along with their derived probiotics, postbiotics, and metabolites, have been increasingly explored. In particular, microbiota-derived metabolites have recently emerged as key mediators of these interactions by influencing immune signaling, inflammation, and stress responses through both systemic circulation and neural pathways such as the vagus nerve.^4^ Bioactive metabolites such as short-chain fatty acids (SCFAs), bile acids, and tryptophan (Trp) catabolites modulate neuroinflammation, reinforce blood–brain barrier integrity, and regulate neurotransmitter production, including serotonin and *γ*-aminobutyric acid.^5-7^

Methylglyoxal (MGO), which is a highly reactive dicarbonyl byproduct generated primarily through non-enzymatic glycolysis and anaerobic microbial fermentation in the large intestine, serves as a major endogenous initiator of carbonyl stress.^8^ Under clinical conditions of chronic hyperglycemia, insulin resistance, or metabolic overload, the excessive accumulation of MGO accelerates the cross-linking of functional cellular proteins, which subsequently triggers the formation of advanced glycation end-products (AGEs).^9^ Accumulating epidemiological and clinical evidence indicates that this MGO/AGEs-driven carbonyl stress is heavily implicated in a broad spectrum of chronic human pathologies, including gastrointestinal epithelial barrier dysfunction, systemic inflammation, and central neuropsychiatric disorders such as major depressive disorder.^9^ ^-11^ These MGO-derived AGEs further promote oxidative stress and neuroinflammation, which directly worsen gut-brain axis dysfunction.^10^ ^,^ ^11^ Furthermore, MGO significantly alters the gut microbial composition and reshapes the intestinal metabolome.^12^ ^,^ ^13^ In particular, this localized intestinal insult severely alters Trp metabolic homeostasis, shifting the catabolic balance away from neuroprotective pathways and accelerating the generation of gut-derived neurotoxins, such as xanthurenic acid *via* the kynurenine pathway and 3-indoxyl sulfate (3-IS) *via* the indole pathway. These neurotoxic metabolites translocate into the systemic circulation and trigger secondary central neuropathology.^14^ ^,^ ^15^ Despite these insights into the pathological role of carbonyl stress in gut-brain axis dysfunction, targeted strategies to modulate MGO/AGEs-related pathways through microbiota-derived interventions remain largely underexplored.

Among such interventions, microbiota-derived metabolites and their functional formulations, known as postbiotics, have emerged as promising candidates for metabolite-targeted modulation of host-microbe interactions.^16^ ^,^ ^17^ However, rational discovery of bioactive metabolites with functional relevance remains challenging. Conventional approaches to probiotic or postbiotic selection rely primarily on phenotypic screening, whereas metabolomic analyses are typically performed retrospectively to identify active compounds, often leading to inconsistent outcomes.^18^ Microbial metabolic signatures exhibit substantial strain-level heterogeneity, making it difficult to predict functional activity based solely on taxonomic classifications.^19^ ^,^ ^20^ Metabolomic-based chemotaxonomy, which classifies biological systems based on their biochemical composition, has been used to identify functional chemotypes and novel bioactive compounds in various biological systems.^21^ ^,^ ^22^ These limitations underscore the need for a systematic metabolomic-derived strategy that can link strain-specific metabolic signatures to functional phenotypes and enable the rational discovery of postbiotic candidates.

In this study, we established a strain-resolved metabolomics framework to enable the rational discovery of bioactive metabolites and the selection of a corresponding postbiotic extract, hypothesizing that a microbial functional chemotype with robust MGO-AGEs breaking activity would yield a candidate capable of modulating gut-brain-related phenotypes under carbonyl stress. Using this approach, we identified LFEAN031, an intracellular metabolite extract enriched with MGO-AGEs breaking products derived from a highly functional *L. fermentum* strain, as a postbiotic candidate. LFEAN031 extract administration significantly alleviated MGO-induced gut-brain pathology, as evidenced by the restoration of intestinal barrier integrity, normalization of hypothalamic-pituitary-adrenal (HPA) axis activation, and improvements to depressive- and anxiety-like behaviors. Cecal metabolomic analyses revealed that these effects were associated with shifts in Trp metabolism toward protective pathways, alongside alterations in *γ*-glutamyl peptide (GGP)-associated redox metabolism. These findings provide a proof-of-concept for a metabolite-guided strategy for postbiotic discovery and highlight the potential of microbiota-derived metabolites as modulators of gut-brain axis function under carbonyl stress.

## Materials and methods

### Bacterial strains, cultivation, and postbiotic extract preparation

A total of 113 bacterial strains were analyzed, including species from four genera within the *Lactobacillaceae* family (*Limosilactobacillus*, *Lactiplantibacillus*, *Lacticaseibacillus*, and *Ligilactobacillus*), two *Enterococcus* species, and four *Bifidobacterium* species (Table 1). Detailed information is provided in Data S1. Strains were cultured in modified De Man, Rogosa, and Sharpe (mMRS; Difco, Sparks, MD, USA) medium supplemented with 0.05% (w/v) L-cysteine (Sigma-Aldrich, St. Louis, MO, USA). Strains were pre-cultured twice in mMRS medium at 37 °C under anaerobic conditions. Cells [optical density (OD)_600_ = 1.0] were inoculated at 1% (v/v) into 50 mL of fresh mMRS broth and incubated at 37 °C for 24 h for the main culture. Anaerobic cultures were maintained in an anaerobic chamber (Coy Laboratory Products, Grass Lake, MI, USA), and aerobic lactic acid bacteria (LAB) cultures were incubated under atmospheric conditions with shaking. Cells were harvested through centrifugation and washed with ice-cold phosphate-buffered saline (PBS). To prepare the postbiotic extract, intracellular metabolites were extracted from the cell pellets by resuspending them in ice-cold acetonitrile:methanol:water (AMW) solvent (40:40:20, v/v/v), followed by three freeze-thaw cycles and bead beating. ^23^ After centrifugation, the supernatant was divided into two aliquots and dried using a vacuum concentrator (Biotron, Pocheon, Korea). The final dry weight of the metabolite extracts was measured, and the extracts were stored at −80 °C until analysis.

**Table 1.** Summary of the 16 probiotic groups used in activity-based metabolomic screening.

Family	Species	Number of strains	Cultivation	Abbreviation (PGCgroup)	Colors
Lactobacillaceae (*n* = 38)	*Ligilactobacillus salivarius*	5	Aerobic	LSAAE (E)	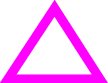
			Anaerobic	LSAAN (E)	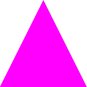
	*Lactiplantibacillus plantarum*	18	Aerobic	LPLAE (D)	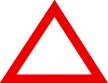
			Anaerobic	LPLAN (D)	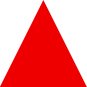
	*Lacticaseibacillus rhamnosus*	5	Aerobic	LRHAE (E)	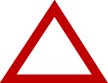
			Anaerobic	LRHAN (E)	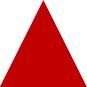
	*Limosilactobacillus fermentum*	10	Aerobic	LFEAE (E)	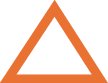
			Anaerobic	LFEAN (E)	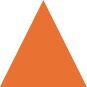
Enterococcaceae (*n* = 26)	*Enterococcus faecalis*	13	Aerobic	EFSAE (C)	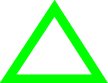
			Anaerobic	EFSAN (C)	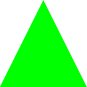
	*Enterococcus faecium*	13	Aerobic	EFMAE (B)	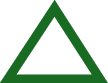
			Anaerobic	EFMAN (B)	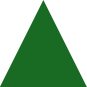
Bifidobacteriaceae (*n* = 49)	*Bifidobacterium animalis* subsp. *lactis*	13	Anaerobic	BANAN (A)	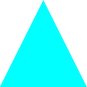
	*Bifidobacterium bifidum*	10	Anaerobic	BBIAN (A)	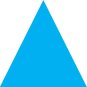
	*Bifidobacterium breve*	13	Anaerobic	BBRAN (A)	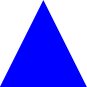
	*Bifidobacterium longum*	13	Anaerobic	BLOAN (E)	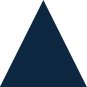

The intracellular metabolite extracts obtained from the 113 strains tested in this study were grouped according to taxonomic identity and culture conditions. Additional information is provided for each group, such as the number of strains, abbreviations, and assigned colors. The assigned colors were used in multivariate analysis in Figure 2.

### Untargeted metabolite profiling of postbiotic extracts

Untargeted metabolomic analysis was performed using gas chromatography-time-of-flight mass spectrometry (GC-TOF-MS) and ultra-high-performance liquid chromatography-Orbitrap tandem mass spectrometry (UHPLC-Orbitrap-MS/MS). The dried metabolite extracts were resuspended in 250 µL of AMW solvent, sonicated for 10 min, and centrifuged at 24,249 × g for 10 min at 4 °C. The supernatant was filtered through a 0.22 μm membrane filter. Subsequently, 80 µL of the resulting clear supernatant was transferred to a new vial for LC-MS analysis, and a separate aliqout dried for GC-MS analysis. All samples were analyzed in randomized order to minimize bias due to instrument drift.

#### LC-MS analysis

For LC-MS analysis, 5 µL of the prepared sample was directly injected into the UHPLC-Orbitrap system. The system comprised the Vanquish Binary Pump H system (Thermo Fisher Scientific, Waltham, MA, USA) coupled with an Orbitrap Velos Pro mass spectrometer equipped with a HESI-II probe. Chromatographic separation was performed on a Kinetex C_18_ column (100 mm × 2.1 mm, 1.7 µm; Phenomenex, Torrance, CA, USA) at 40 °C. The mobile phases were 0.1% (v/v) formic acid in water (solvent A) and 0.1% (v/v) formic acid in acetonitrile (solvent B). The percentage of solvent B was maintained at 5% for 1 min, linearly increased to 100% over the next 9 min, maintained at 100% for 1 min, and finally decreased to 5% over 3 min to re-equilibrate the column. Full-scan MS data were acquired in the m/z range of 100–1,500 in negative ion mode using Orbitrap. The HESI probe heater and capillary temperature were set to 300 °C and 350 °C, respectively, and the capillary voltage was set to −2.5 kV.^24^

#### GC-MS analysis

The dried residue was subjected to two-step derivatization. First, oximation was conducted by adding 40 µL of methoxyamine hydrochloride in pyridine (20 mg/mL) and incubating at 30 °C for 90 min. Subsequently, silylation was performed by adding 40 µL of N-methyl-N-(trimethylsilyl)trifluoroacetamide (MSTFA) and incubating at 37 °C for 30 min. The derivatized sample (1 µL) was injected into an Agilent 7890B GC system coupled to a Pegasus BT TOF-MS (LECO, St. Joseph, MI, USA) with a 10:1 split ratio. The injector temperature was maintained at 250 °C. Chromatographic separation was performed on a Rtx-5MS capillary column (30 m × 0.25 mm, 0.25 μm film thickness; Restek, Bellefonte, PA, USA) using helium as the carrier gas at a constant flow rate of 1.5 mL/min. The column oven was initially held at 300 °C for 3 min. The transfer line temperature was set to 240 °C, and mass spectra were acquired under electron ionization at 70 eV over a mass range of m/z 50–600.^25^

#### Data processing

Raw data files from GC-TOF-MS and UHPLC-LTQ-Orbitrap-MS/MS were converted to NetCDF (.cdf) formats using LECO ChromaTOF software (v.4.44) and the ABF format using Analysis Base File Converter. Retention time correction, peak detection, and alignment were performed using MetAlign software package (http://www.metalign.nl) for GC-MS data and MS-dial software (https://xcmsonline.scripps.edu) for LC-MS data. The aligned datasets were merged and normalized to the dry weight of intracellular metabolite extracts for multivariate analyses, including principal component analysis (PCA), partial least squares discriminant analysis (PLS-DA), and hierarchical clustering analysis (HCA).

### MGO-AGEs breaking activity screening of postbiotic extracts

MGO-modified bovine serum albumin (MGO-AGEs) was prepared by incubating bovine serum albumin (BSA; 5 mg/mL) with MGO (10 mM) and 0.02% sodium azide in PBS (pH 7.4) at 37 °C for 7 d. The resulting mixture was concentrated, desalted using Zeba Spin Desalting Columns (7 kDa MWCO; Thermo Fisher Scientific, Waltham, MA, USA), and lyophilized. AGEs-breaking activity was assessed using a modified 2,4,6-trinitrobenzene sulfonic acid (TNBSA) assay.^26^ Briefly, MGO-AGEs (0.5 mg/mL) were mixed with intracellular metabolite extracts (250 µg/mL) and incubated at 37 °C for 24 h. Subsequently, 4% sodium bicarbonate (pH 8.5) and 0.1% TNBSA were added, followed by incubation at 37 °C for 2 h. The reaction was terminated by adding 10% sodium dodecyl sulfate (SDS), and 1 N hydrochloric acid was added and vortexed. The optical absorbance of the mixture was measured at 340 nm using a microplate reader (Molecular Devices, San Jose, CA, USA).

### Chemotype-guided prioritization of bioactive compounds and postbiotic candidates

#### Metabolomic-based chemotaxonomy

Multivariate analyses were performed using SIMCA (v.15.0.2; Umetrics, Umea, Sweden), based on the combined metabolomic datasets obtained from both GC-MS and LC-MS. HCA was used to visualize metabolic relationships between groups. For species-level analysis, HCA was conducted on the PLS-DA scores to assess group-discriminating features, whereas for strain-level analysis, HCA was performed on the PCA scores to investigate unbiased intrinsic clustering. Chemotypes were delineated by segmenting dendrograms at defined distance thresholds, and their functional relevance was evaluated using one-way analysis of variance (ANOVA). Discriminant metabolites among chemotype groups were identified based on variable importance in projection (VIP) values from the PLS-DA model and significance (*p* value < 0.05) (Data S2 and S3).

#### *In vitro* validation of bioactive compounds

Potential MGO-AGEs breaking compounds from the strain-level functional chemotype were defined based on three criteria: (1) discriminant metabolites among chemotype groups, (2) a fold change (FC) > 2.0 in abundance in high-activity chemotype groups (LFCB) compared with that in other clusters, and (3) a significant positive correlation with MGO-AGEs breaking activity. Based on these criteria, five candidate compounds were selected, including Trp, asparagine (Asn), and three GGPs. Authentic standards of the three GGPs were synthesized by Peptron (Daejeon, Korea), and Trp and Asn were purchased from Sigma-Aldrich (St. Louis, MO, USA). These standards were used to achieve MSI level 1 identification by LC-MS and GC-MS analysis, based on matching retention times and MS/MS spectra (LC-MS) or retention indices and EI mass spectra (GC-MS) under identical conditions (Data S2-S3), and to validate their *in vitro* MGO-AGEs breaking activity using the TNBSA assay, as described above. Briefly, MGO-AGEs (0.5 mg/mL) were incubated with each test compound (50, 100, and 200 µM) at 37 °C for 24 h. Following incubation, the TNBSA reaction, termination, and absorbance measurements at 340 nm were performed according to an established protocol.

#### Selection of lead postbiotic candidate

The leading postbiotic candidate for *in vivo* validation was selected using a multi-criteria framework integrating chemometric profiling with functional activity. Selection was based on three criteria: (1) strains within the chemotype exhibiting the highest mean MGO-AGEs breaking activity, (2) the highest individual activity within that chemotype cluster, and (3) enrichment of identified bioactive metabolites, including Trp, Asn, and GGPs, as determined using chemotype-based clustering. Based on these criteria, LFEAN031 (derived from *L. fermentum* KCTC 15072BP) was selected for subsequent evaluation in a murine model.

### *In vivo* evaluation of the selected postbiotic extract in an MGO-induced gut-brain dysfunction model

#### Animals and experimental design

Institute of Cancer Research (ICR) mice (7-week-old, male) were obtained from Orient Bio Inc. (Gyeonggi-do, Korea) and acclimated for 1 week under a 12/12 h light/dark cycle at 23 ± 1 °C and 60 ± 5% humidity prior to the start of experiments. The mice were fed a laboratory diet (AIN-76A) and provided with water *ad libitum*. After adaptation, the mice were randomly divided into three groups (six mice per group): control (Ctrl), MGO-treated (MGO, 60 mg/kg, twice weekly), and MGO co-treated with LFEAN031 (LFEAN031, 50 mg/kg/d); for brevity, this MGO + LFEAN031 co-treatment group is denoted “LFEAN031” throughout. MGO (dissolved in 30% v/v glycerol in PBS, pH 7.4) (Sigma-Aldrich, St. Louis, MO, USA) was administered to experimental ICR mice for 2 weeks (twice per week) at 60 mg/kg *via* rectal injection. The rectal route of administration was specifically utilized to closely mimic the clinical pathophysiology of gut-derived carbonyl stress, as MGO is predominantly generated *via* anaerobic microbial fermentation within the luminal environment of the large intestine. Furthermore, this targeted localized delivery bypasses potential chemical degradation or neutralization by gastric acids and upper gastrointestinal enzymes, thereby allowing a direct and controlled challenge to colonic barrier integrity as assessed by ZO-1 and the subsequent evaluation of leaky gut-mediated gut-brain axis disruption, consistent with our previously validated *in vivo* models.^27^ LFEAN031 was dissolved in saline and orally administered daily for 2 weeks. Mice exhibiting clinical abnormalities or ≥20% weight loss were humanely euthanized using CO_2_ (30–70% volume/min; AVMA guidelines) with death confirmed after ≥5 min of respiratory and cardiac arrest, under approval by the Institutional Animal Care and Use Committee of Gachon University (Incheon, Korea; Approval No. GU1-2022-IA0046).

#### Depression-like behavior tests

The open field test (OFT), tail suspension test (TST), and forced swimming test (FST) were conducted to evaluate locomotor activity and anxiety- and depression-like behaviors in a mouse model of MGO-induced depression. In the OFT, mice were placed in the center of a black arena (60 × 60 × 60 cm) and monitored for 5 min using SMART v.3.0 video tracking system (Panlab; Harvard Apparatus, Barcelona, Spain) to assess the time spent in the central zone and the total distance traveled. For the TST, each mouse was suspended by the tail in a chamber (60 cm height × 15 cm width × 11.5 cm depth) using adhesive tape, and immobility time was recorded for 4 min after a 2-min acclimation. In the FST, mice were placed in a transparent cylinder (50 cm height × 20 cm diameter) filled with 30 cm of water at room temperature. After 2 min of adaptation, immobility time was measured over a 4-min test period using SMART v.3.0 system.

#### Enzyme-linked immunosorbent assay (ELISA) analysis

Serum levels of pro-inflammatory cytokines IL-6, the anti-inflammatory cytokine IL-10, and cortisol were measured using ELIZA kits (R&D Systems, Minneapolis, MN, USA) following the manufacturer’s instructions. Quantification was performed using colorimetric detection.

#### Histological and immunohistochemical analysis

For histological analysis, mouse colonic tissues were fixed in 10% neutral-buffered formalin, dehydrated using graded ethanol, cleared in xylene, and embedded in paraffin. Sections (4 µm) were cut and stained with hematoxylin and eosin following standard procedures. The stained slides were examined at 100 × magnification using Nikon Eclipse 80i microscope (Nikon, Tokyo, Japan), and quantitative analysis was performed using ImageJ software (NIH, Bethesda, MD, USA). Immunohistochemical analysis was conducted to evaluate glucocorticoid receptor (GR) expression in the hypothalamic paraventricular nucleus (PVN) (bregma −0.70 to −0.90 mm) and zonula occludens-1 (ZO-1) in the large intestine.^27^ Sections were treated with 1% hydrogen peroxide, blocked in PBS containing 3% normal goat serum and 1% BSA, and incubated overnight at 4 °C with rabbit anti-GR antibody (1:500, Santa Cruz Biotechnology, Dallas, TX, USA) and rabbit anti-ZO-1 antibody (1:200, Abcam, Cambridge, UK) in PBS with 0.3% Triton X-100. After incubation with biotinylated goat anti-rabbit IgG and avidin-biotin complex, DAB was used for visualization. The slides were mounted with DPX and imaged at 100× magnification using a Nikon Eclipse 80i microscope (Nikon).

### Metabolomic analysis of cecal contents and brain tissues

#### Untargeted metabolomics

Cecal contents and brain tissue (50 mg) were mixed with 20 volumes of 50% methanol containing 2-chloro-phenylalanine as an internal standard (10 μg/mL).^28^ The mixture was subjected to bead beating and sonication. After centrifugation, the microfiltered (0.22 μm) supernatant was collected, dried to 0.3 mL under vacuum, and then reconstituted in 0.8 mL of 50% MeOH solvent. The filtered samples were loaded into new vials for LC-Orbitrap-MS analysis. The UHPLC system was integrated with Vanquish binary pump C system coupled to an Orbitrap Exploris 120 mass spectrometer (Thermo Fisher Scientific) for untargeted analysis of MGO-AGEs breaking compounds.^29^ Chromatographic separation was conducted using Atlantis Premier BEH Z-HILIC Column (100 mm × 2.1 mm, 1.7 μm particle size; Waters, Wexford, Ireland). Mobile phase A comprised 10 mM ammonium acetate in acetonitrile:water (10:90, v/v), adjusted to pH 9.05 with ammonium hydroxide. Mobile phase B was 10 mM ammonium acetate in acetonitrile:water (90:10, v/v), adjusted to pH 9.05 with ammonium hydroxide. The percentage of solvent B was maintained at 5% for 1 min, linearly increased to 100% over the next 9 min, maintained at 100% for 1 min, and finally decreased to 5% over 3.5 min to re-equilibrate the column. Full-scan MS data were acquired in the m/z range of 70–1,000 in negative ion mode using Orbitrap. Raw data were processed using the same pipeline as that used for the probiotic extract dataset. The aligned datasets were normalized to the internal standard and log_2_-transformed. PCA was performed to visualize global metabolic differences among the samples. Multivariate permutational ANOVA (PERMANOVA) was performed using 999 permutations to assess the statistical significance of the group separation observed in the PCA score plots. Euclidean distances were calculated from the PCA score coordinates, and statistical testing was conducted using MetaboAnalyst 6.0. The recovered metabolites were defined as those exhibiting reversal trends under postbiotic treatment relative to carbonyl stress. Metabolites were selected based on the following criteria: (1) statistical significance among groups (one-way ANOVA, *p* < 0.10) and (2) directional recovery, defined as a greater relative change between the postbiotic-treated and MGO groups than that observed between the Ctrl and MGO groups [log_2_(LFEAN031/MGO) > 0.5 × log_2_(MGO/Ctrl)]. The relative abundances of the recovered metabolites were visualized using autoscaled heatmaps and bar graphs.

#### Targeted metabolomics of tryptophan-related pathways

Targeted metabolomics was conducted on 32 compounds involved in Trp metabolism, including 1 precursor, 12 indole (IND) pathway metabolites, 12 kynurenine pathway metabolites, and 7 serotonin pathway metabolites. Sample extracts prepared for untargeted metabolomics were analyzed using Nexera X2 LC system (Shimadzu Corp., Kyoto, Japan) combined with a triple-quadrupole MS equipped with an electrospray source (LCMS-8040; Shimadzu).^30^ Chromatographic separation was achieved using Waters ACQUITY UPLC HSS T_3_ column (150 mm × 2.1 mm, 1.8 μm particle size; Waters, Wexford, Ireland). The mobile phase consisted of 0.1% formic acid in water (solvent A) and 0.1% formic acid in acetonitrile (solvent B). The percentage of solvent B was maintained at 0% for 1 min, linearly increased to 28% over the next 7 min, further increased to 100% over the next 0.5 min, held at 100% for 3 min, and finally decreased to 0% over 4 min to re-equilibrate the column. Detailed information concerning the Trp-related compounds with multiple reaction monitoring transitions is provided in Data S4. Comparative analysis was performed by manually extracting the peak areas of each identified compound from the raw data using instrument-specific software, such as FreeStyle and LabSolutions. Peak areas were normalized to the internal standard for relative quantification across different treatment groups.

### Statistical analysis

All statistical analyses were performed using GraphPad Prism 10 software (GraphPad Software Inc., San Diego, CA, USA). ANOVA followed by Tukey’s *post hoc* test was used for multiple group comparisons (microbial groups and *in vivo* outcomes). Pearson’s correlation analysis was performed to determine the relationship between metabolite abundance and MGO-AGEs breaking activity. *In vitro* validation of candidate MGO-AGEs breaking compounds was conducted using one-way ANOVA, followed by Dunnett’s *post hoc* test. All data are presented as means ± standard deviation (SD) with individual data points and biological replicates shown where applicable.

## Results

### Strain-resolved metabolomics enables functional classification of MGO-AGEs breaking chemotypes

**Figure 1.**
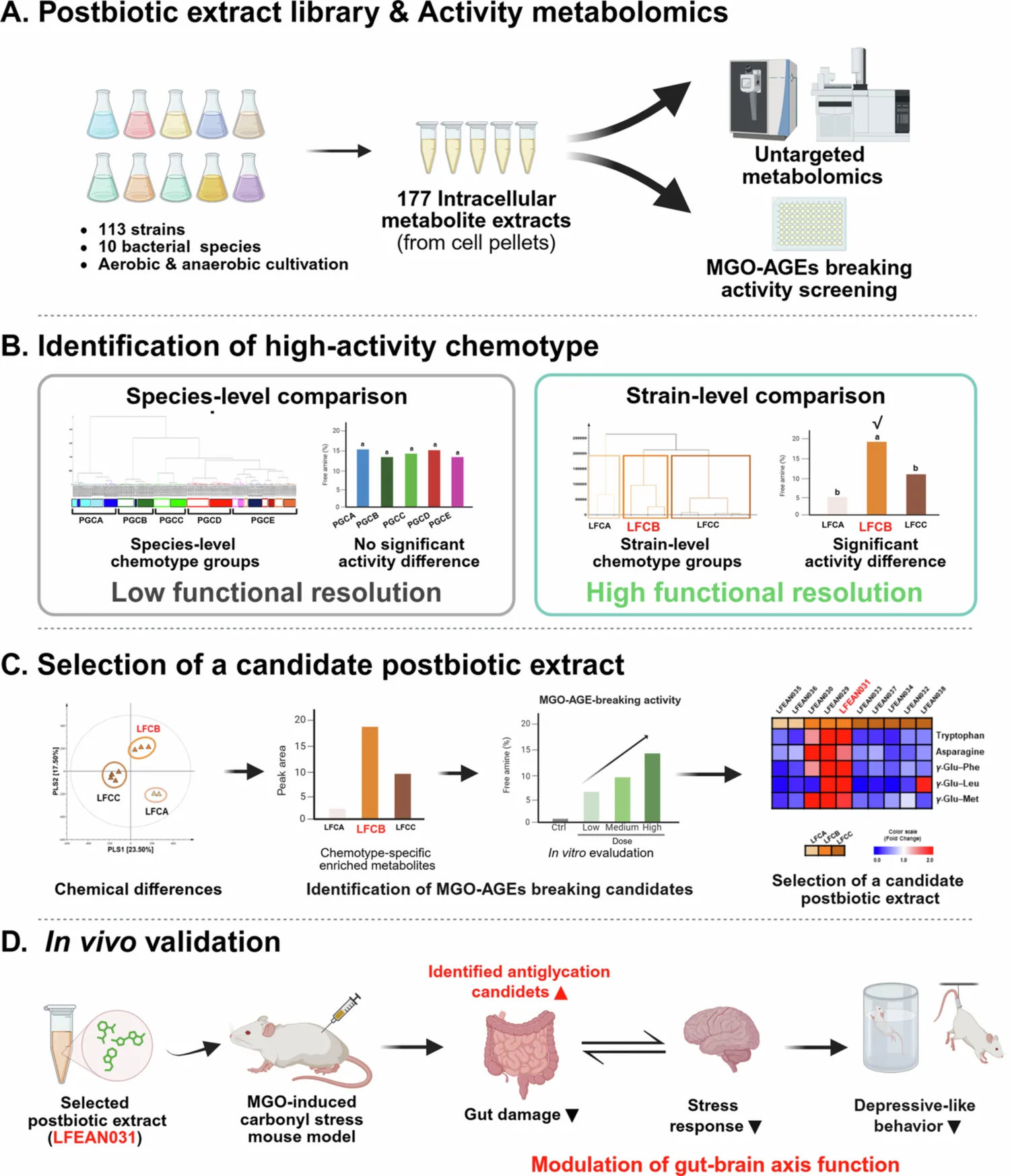
Strain-resolved activity metabolomics framework prioritizes a postbiotic extract, LFEAN031, by active metabolite-guided rational selection. (A) Schematic overview of postbiotic extract library construction and activity-based metabolomic profiling. A total of 113 bacterial strains across 10 species were individually cultured under aerobic and/or anaerobic conditions, yielding 177 intracellular microbial metabolite extracts that collectively formed the postbiotic extract library. Each extract was subjected in parallel to untargeted metabolomics and an *in vitro* MGO-AGEs breaking activity screen. (B) Comparison of functional resolution between species-level and strain-level chemotype classification. Hierarchical clustering (HCA) based on metabolite profiles defined five species-level chemotype groups (PGCA−PGCE), which showed no significant differences in MGO-AGEs breaking activity, indicating low functional resolution. Within the *L. fermentum*/anaerobic (LFEAN) postbiotic group, strain-level chemotype groups (LFCA−-LFCC) were observed, with LFCB showing the highest activity. (C) Selection of the candidate postbiotic extract. LFCB-specific enriched metabolites were identified using a PLS-DA model and functionally validated *in vitro*. LFEAN031 was selected based on its enrichment of marker compounds and its highest MGO-AGEs breaking activity among LFEAN strains classified as LFCB. (D) *In vivo* validation of the selected postbiotic extract LFEAN031. LFEAN031 was orally administered in an MGO-induced carbonyl stress mouse model. LFEAN031 attenuated gut damage and stress responses, and reduced depressive-like behaviors, accompanied by increased levels of identified marker metabolites in cecal contents. Figure 1 was created using BioRender (https://BioRender.com/gxhqcia).

**Figure 2.**
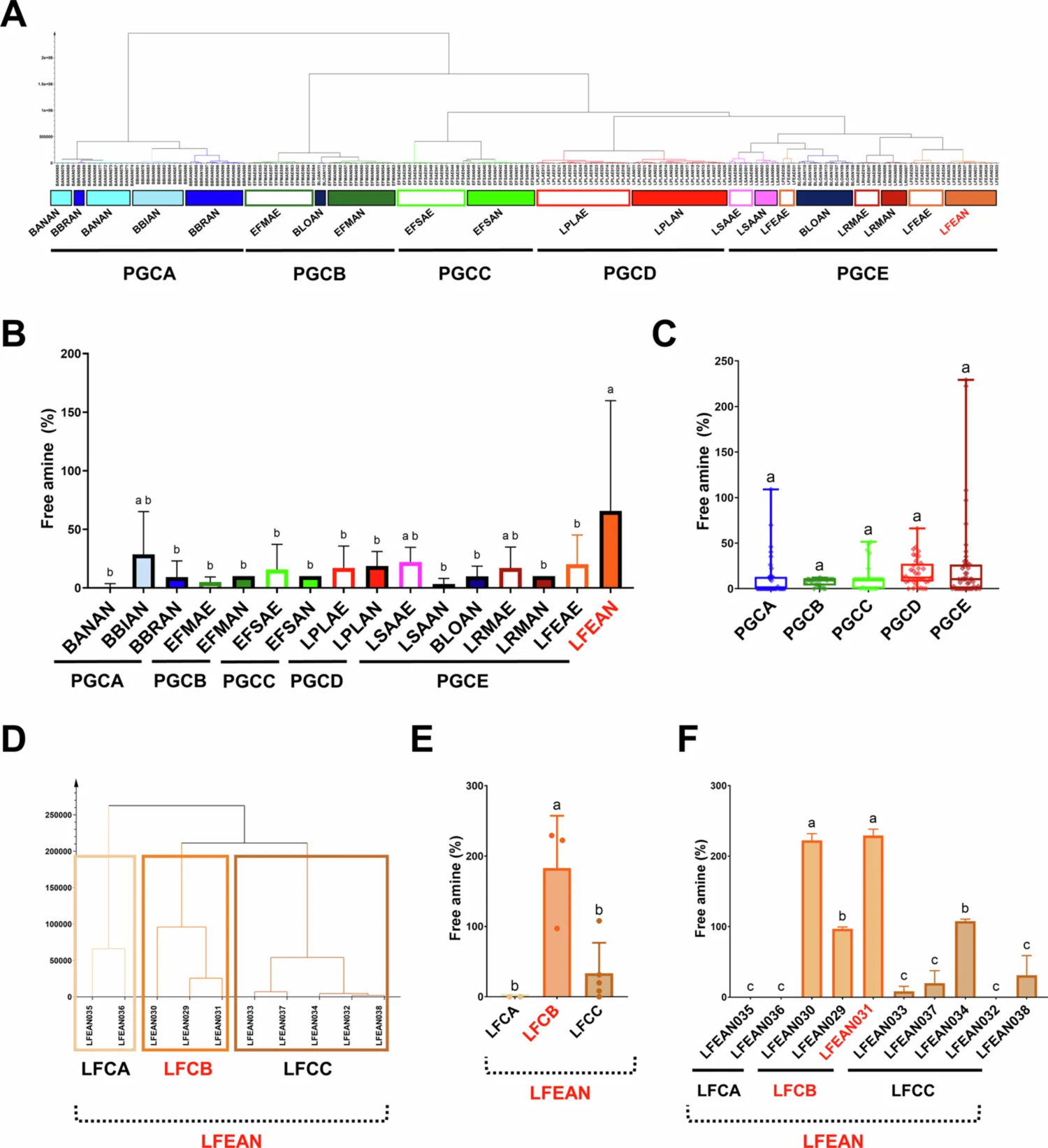
Strain-resolved activity metabolomics framework identifies high MGO-AGEs breaking activity chemotypes. (A) Hierarchical clustering analysis (HCA) dendrogram of the 16 postbiotic groups based on the partial least squares discriminant analysis (PLS-DA) result of metabolite profiles. These groups were classified into five species-level clusters (distance threshold of 7 × 10^5^), designated PGCA–PGCE. (B, C) Antiglycation activity of 16 postbiotic groups (B) and five species-level chemotypes (C) using MGO-AGEs breaking assay. (D) HCA dendrogram of LFEAN postbiotic extracts based on principal component analysis (PCA) distances. The 10 anaerobically cultured *Limosilactobacillus fermentum* strains were classified into three strain-level clusters (distance threshold of 1.5 × 10^6^), designated LFCA–LFCC. (E, F) Antiglycation activity of three strain-level chemotypes (E) and 10 anaerobically cultured *L. fermentum* strains (F) using MGO-AGEs breaking assay. Different letters in panels B-C and E-F denote statistically significant differences among 16 postbiotic groups, chemotype groups, and individual LFEAN extracts (*p* < 0.05, one-way ANOVA with Tukey’s *post hoc* test). Data are presented as means ± standard error of the mean (mean ± SEM).

To systematically explore microbial chemotypes associated with MGO-AGEs breaking activity, we applied a strain-resolved activity metabolomics framework to a postbiotic extract library (Figure 1A−D). This library comprised 177 intracellular metabolite extracts, individually prepared from 113 strains across 10 probiotic species, cultured under both aerobic and anaerobic conditions (Figure S1, Data S1). For comparative analysis, these 177 extracts were subsequently grouped into 16 postbiotic groups based on taxonomy and cultivation conditions (Table 1). These extracts were screened using untargeted metabolomics and *in vitro* MGO-AGEs breaking assay (Figure 1A, Data S1). The metabolic profiles displayed distinct clustering patterns that corresponded more closely to taxonomic identities at the family and species levels than to culture conditions (Figures 2A, S2A–S2C, Data S5).

Given that clustering patterns were primarily driven by taxonomic identity, we next investigated whether MGO-AGEs breaking activity could be resolved by grouping postbiotic extracts into species- and strain-level chemotypes. Five species-level chemotypes (PGCA–PGCE) were defined based on HCA dendrogram distances, reflecting divergent chemical characteristics (Figures 2A, S2D, Table S1). Despite the robust chemical clustering of chemotypes, their MGO-AGEs breaking activities exhibited no significant differences and varied markedly within clusters (Figure 2B, C). These results indicate that species-level identity alone is insufficient for predicting functional activity.

To improve functional resolution, we investigated strain-level functional classification, focusing on the LFEAN group (*L. fermentum* metabolite extracts under anaerobic cultivation). This group exhibited both the highest MGO-AGEs breaking activity and the greatest functional heterogeneity among all groups (Figure 2B). Multivariate analyses chemically defined three distinct metabolic clusters (LFCA, LFCB, and LFCC) based on HCA performed on PCA scores, with cluster assignments determined from dendrogram distances (Figures 2D, S3A, Table S2). These chemotypes exhibited significantly different MGO-AGEs breaking activities across clusters, yet maintained functional similarities within each cluster (Figure 2E, F). These findings demonstrate that strain-level metabolic profiling resolves microbial heterogeneity, enabling functional classification beyond species-level resolution.

### Identification of MGO-AGEs breaking compounds enables rational selection of a postbiotic candidate

**Figure 3.**
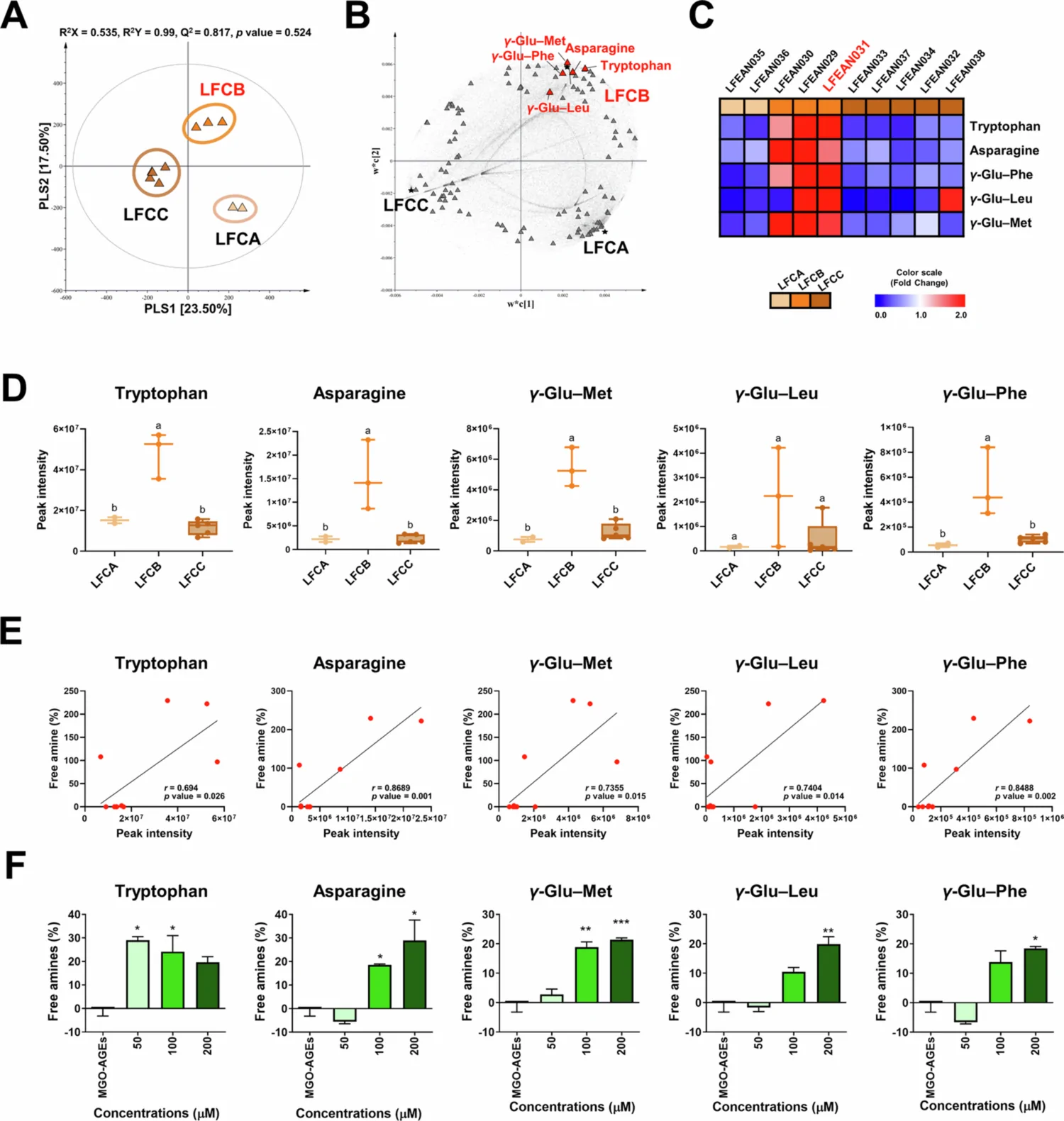
Identification of MGO-AGEs breaking metabolites enriched in high-activity chemotypes. (A) Partial least squares discriminant analysis (PLS-DA) score plot of the three *Limosilactobacillus fermentum* chemotypes (LFCA–LFCC) classified in the hierarchical clustering analysis (HCA) dendrogram. (B) PLS-DA loading plot illustrating LFCB-specific marker compounds among the selected discriminant metabolites. Marker compounds are indicated in red text. (**C)** Heatmap comparing the relative abundance of five LFCB-specific marker compounds across 10 *L. fermentum* strains. The strain selected as postbiotic candidate is indicated in red text. (D) Box plot comparing the relative abundance of the five marker compounds in LFCB across three strain-cluster groups. (E) Correlation and linear regression analyses between MGO-AGEs breaking activity and peak area of LFCB-marker compounds. (F) *In vitro* MGO-AGEs breaking activity fo the five marker compounds. Different letters indicate statistically significant differences among chemotype groups (*p* < 0.05) and asterisks indicate statistical significance between each cluster group and the Ctrl group (*p < 0.05, **p < 0.01, ***p < 0.001, one-way ANOVA with *post hoc* test). Error bars represent standard deviation. *γ*-Glu-Met, *γ*-Glutamyl-methionine; *γ*-Glu-Leu, *γ*-Glutamyl-leucine; *γ*-Glu-Phe, *γ*-Glutamyl-phenylalanine.

Building on the strain-resolved functional classification, we identified key MGO-AGEs breaking metabolites associated with chemotypes exhibiting high MGO-AGEs breaking activity. PLS-DA was performed on LFEAN extracts from the *L. fermentum* strains. The PLS-DA score plot revealed distinct chemical differences among the groups (LFCA, LFCB, and LFCC), consistent with the HCA-based classification (Figure 3A). Discriminant metabolites were selected based on the VIP values (>1.0) from the PLS-DA model, and chemotype-specific marker compounds were defined using an FC criterion (FC > 2.0) (Data S6). The LFCB group, which exhibited the highest MGO-AGEs breaking activity, was enriched in amino acids and their derivatives compared with those of the other groups (Figure S3B). In particular, five LFCB-enriched marker metabolites were identified: two amino acids (Trp and Asn) and three GGPs [*γ*-glutamyl-methionine (*γ*-Glu-Met), *γ*-glutamyl-phenylalanine (*γ*-Glu-Phe), and *γ*-glutamyl-leucine (*γ*-Glu–Leu)] (Figures 2B, 3B−D).

Correlation analysis and *in vitro* validation were performed to evaluate the MGO-AGEs breaking potential of chemotype-enriched marker metabolites. All five metabolites displayed strong positive correlations (*r* = 0.69–0.87) with MGO-AGEs breaking activity and exhibited concentration-dependent effects, confirming their functional relevance (Figure 3E, 3F). Among them, Trp displayed the most potent MGO-AGEs breaking activity at a concentration of 50 µM, and the activity tended to decrease at higher concentrations. The MGO-AGEs breaking activities of the other four compounds increased with increasing concentration. The MGO-AGEs breaking activity measured at 200 μM followed the order: Asn > *γ*-Glu-Met > *γ*-Glu-Leu > *γ*-Glu-Phe.

These results suggest that strain-level metabolomic profiling can link MGO-AGEsbreaking activity to specific microbial chemotypes and their associated metabolic signatures, thereby providing a rational basis for the selection of postbiotic candidates.

### LFEAN031 postbiotic extract attenuates MGO-induced intestinal barrier damage, systemic stress, and neurobehavioral deficits

Building on these findings of strain-resolved functional metabolomics, we rationally selected LFEAN031 as the postbiotic extract for *in vivo* evaluation using multi-criteria selection. LFEAN031 is an intracellular metabolite extract derived from *L. fermentum* KCTC 15072BP that belongs to the high-activity LFCB chemotype, exhibits the highest individual MGO-AGEs breaking activity, and is selectively enriched in the identified bioactive metabolites, including Trp, Asn, and GGPs (Figures 2F, 3C).

**Figure 4.**
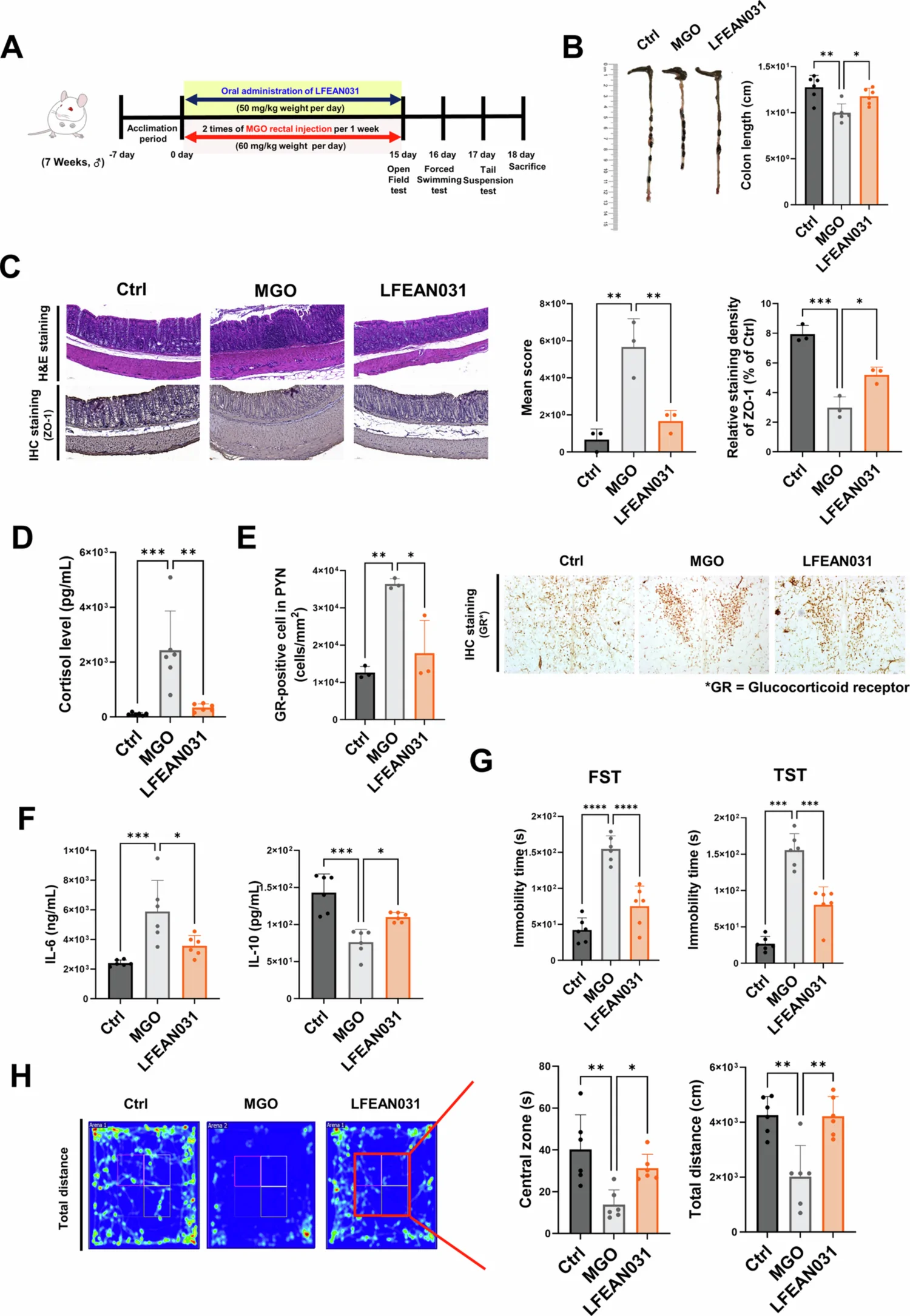
LFEAN031 postbiotic extract attenuates intestinal barrier damage, systemic stress, and neurobehavioral deficits under MGO-induced carbonyl stress. (A) Experimental scheme illustrating oral administration of postbiotic extract (LFEAN031 50 mg/kg/d) for 14 consecutive days following the induction of carbonyl stress *via* rectal injections of MGO (60 mg/kg, twice per week). (B) Representative images and quantification of colon length, demonstrating MGO-induced colon shortening and its recovery following LFEAN031 treatment. (C) Representative histological images (H&E and IHC staining) of colonic tissue and quantification of histological scores and tight junction protein zonula occludens-1 (ZO-1) expression. (D) Serum levels of cortisol. (E) Representative histological images (IHC staining) of the hypothalamic paraventricular nucleus (PVN) of glucocorticoid receptor (GR) expression. (F) Serum levels of pro- and anti-inflammatory cytokines (IL-6 and IL-10, respectively). (G) Total immobility time in the forced swimming test (FST) and tail suspension test (TST). (H) Representative heatmap images and quantification of time spent in the center zone and total distance traveled in the open field test (OFT), reflecting improvements in anxiety-like behavior. Data are presented as means ± standard error of the mean (mean ± SEM, *n* = 3–6). Asterisks indicate statistically significant differences compared with the MGO group (*p < 0.05, **p < 0.01, ***p < 0.001; one-way ANOVA followed by *post hoc* test).

To validate the *in vivo* efficacy of metabolite-guided postbiotic candidates, we evaluated LFEAN031 in a mouse model of MGO-induced carbonyl stress. The LFEAN031 postbiotic extract was orally administered at a dose of 50 mg/kg/d for 14 consecutive days to ICR mice exposed to glycation-induced systemic and neurobehavioral stress *via* rectal injections of MGO (60 mg/kg/d, twice weekly) (Figure 4A). This model was established to induce glycation-associated metabolic dysregulation, and MGO treatment resulted in intestinal barrier damage, dysregulated stress signaling, and depressive- and anxiety-like behaviors.^31^ ^,^ ^32^

Intestinal morphological and barrier function analyses were performed to assess the gut-protective effects of the LFEAN031 extract. MGO administration significantly shortened the colon length, whereas LFEAN031 treatment restored it to control levels (Figure 4B). Immunohistochemical analysis demonstrated that MGO markedly reduced the expression of the tight junction protein ZO-1, whereas LFEAN031 treatment restored its expression, suggesting improved gut barrier integrity (Figure 4C). Next, we examined systemic stress and inflammatory responses. MGO administration significantly increased serum cortisol levels and elevated GR expression in the PVN of the hypothalamus. In contrast, LFEAN031 treatment significantly attenuated these levels, suggesting mitigation of HPA axis hyperactivation induced by carbonyl stress (Figure 4D, E). Furthermore, the inflammatory response was attenuated following LFEAN031 treatment, as evidenced by decreased IL-6 and increased IL-10 levels (Figure 4F).

Consistent with the improvement of gut barrier integrity, HPA axis signaling, and inflammatory responses, we evaluated behavioral outcomes to determine whether these physiological improvements translated into reduced depressive- and anxiety-like behaviors. MGO-treated mice exhibited pronounced depressive-like behaviors, including increased immobility time, in the FST and TST, whereas LFEAN031 treatment significantly reduced immobility in both tests (Figure 4G). In the OFT, the MGO group exhibited a reduced total distance traveled, along with reduced time in the central zone, consistent with anxiety-like behavior. LFEAN031 treatment restored exploratory behavior to levels comparable to those of non-stressed control mice (Ctrls), suggesting a reduction in anxiety-like behavior induced by carbonyl stress (Figure 4H). These findings demonstrate that LFEAN031 alleviates MGO-induced carbonyl stress and its associated intestinal barrier dysfunction, HPA axis and inflammatory dysregulation, and depressive- and anxiety-like behaviors.

### LFEAN031 postbiotic extract modulates MGO-induced intestinal metabolic dysregulation

**Figure 5.**
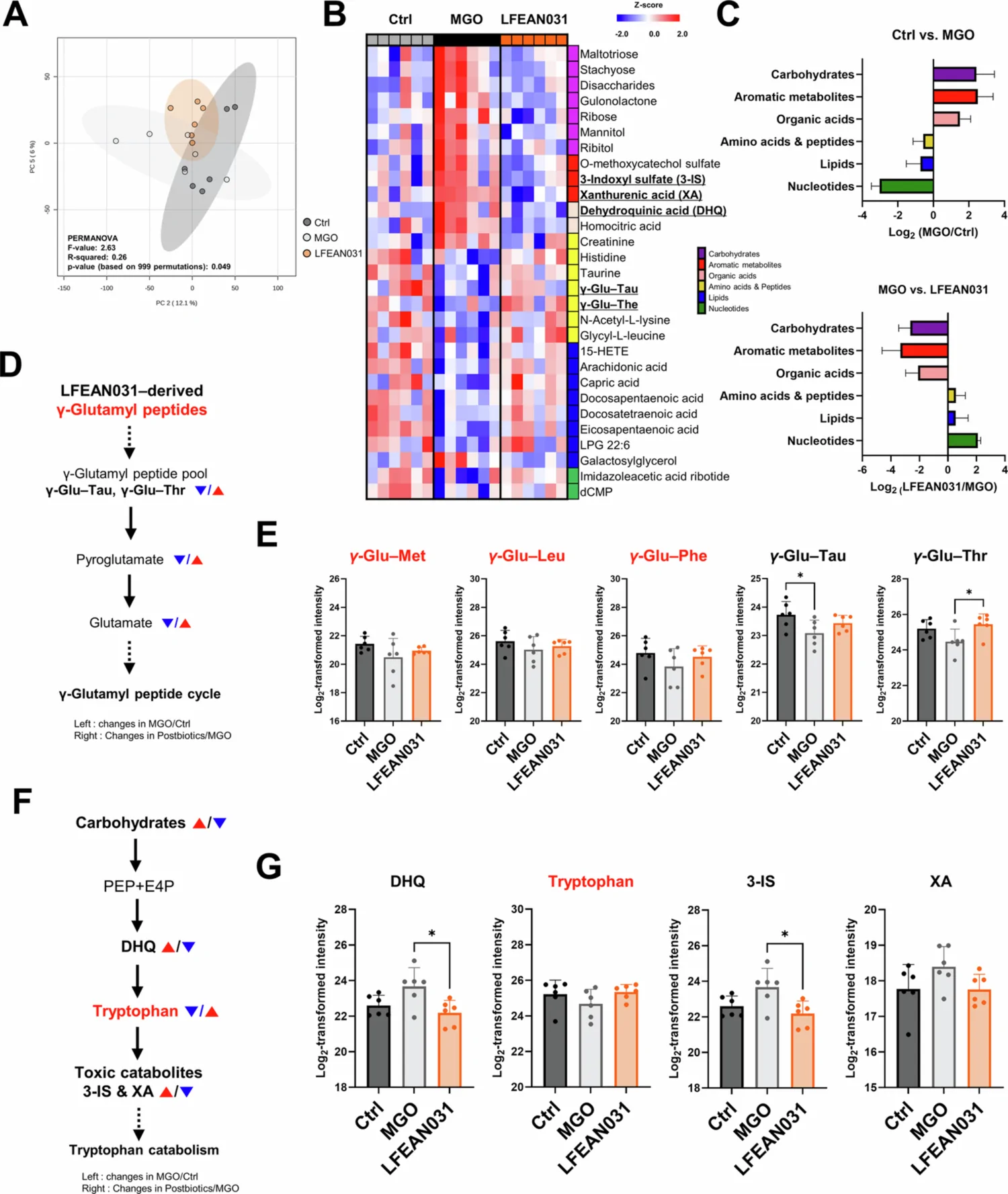
Postbiotic extract LFEAN031 modulates the global intestinal metabolome, particularly *γ*-glutamyl peptide (GGP) cycle and microbial aromatic metabolism. (A) Principal component analysis (PCA) score plot showing global separation of cecal metabolic profiles across groups. (B, C) Heatmaps (B) and bar graph (C) showing restoration of 29 methylglyoxal (MGO)-dysregulated metabolites and their chemical classes following LFEAN031 administration. (D, F) Schematic representations of LFEAN031-induced recovery of metabolic alterations in GGPs (D) and microbial aromatic metabolism (F) under carbonyl stress. Red and blue triangles denote increased and decreased metabolite levels, respectively. Left and right arrows indicate the MGO/Ctrl and postbiotic/MGO comparisons, respectively. (E) Relative abundances of recovered metabolites involved in GGP cycle. (G) Relative abundances of recovered metabolites involved in microbial aromatic metabolism. Antiglycation compounds identified in functional chemotypes are indicated in red text. Asterisks indicate statistically significant differences compared with the MGO group (*p < 0.05, **p < 0.01, ***p < 0.001; one-way ANOVA followed by *post hoc* test).

To determine whether the physiological improvements observed under carbonyl stress were associated with alterations in intestinal metabolism, we performed untargeted metabolomic profiling of the cecal contents. Based on PCA score distances, PERMANOVA revealed significant differences among the groups (F = 2.63, R^2^ = 0.26, *p* = 0.049) (Figures 5A, S4A). These results indicate that carbonyl stress was associated with perturbations in global intestinal metabolic profiles, which were partially modulated following LFEAN031 treatment. We identified 29 metabolites that exhibited recovery-like patterns of MGO-induced dysregulation following LFEAN031 treatment (Figure 5B and Data S7). Carbohydrates, aromatic metabolites, and organic acids generally increased under MGO exposure (positive log_2_[MGO/Ctrl] and decreased following LFEAN031 treatment (negative log2[LFEAN031/MGO]), whereas amino acids, lipids, and nucleotide-related metabolites showed the opposite trend (Figures 5C, S4B).

We next examined whether the five MGO-AGEsbreaking candidate metabolites used to select LFEAN031 were reflected in cecal metabolome changes following postbiotic treatment. Interestingly, LFEAN031 administration showed increasing trends in these five marker compounds and recovery-like shifts in metabolites belonging to the GGP pool and the aromatic axis (Figures 5D−G, S4C). Among the recovered metabolites, *γ*-glutamyl–taurine (*γ*-Glu–Tau) and *γ*-glutamyl-threonine (*γ*-Glu-Thr) showed decreased abundance under MGO exposure and increased abundance following LFEAN031 treatment, and the three bioactive GGP candidates exhibited similar directional changes (Figures 5D, E, S4D).

In parallel, MGO administration led to marked accumulation of carbohydrate-related metabolites, including maltotriose, stachyose, and aromatic metabolites, together with increased levels of Trp, indicating impaired microbial utilization and dysbiosis (Figure 5F).^33^ In particular, altered metabolic shifts were observed in the shikimate pathway, characterized by the accumulation of dehydroquinic acid (DHQ) and a subsequent increase in toxic metabolites, including 3-indoxyl sulfate (3-IS) and xanthurenic acid (XA) (Figure 5G).^34^ ^,^ ^35^ Together, these findings suggest that the local bioavailability of the identified MGO-AGEs breaking metabolites is associated with modulation of the intestinal metabolic pool relevant to MGO-AGEs breaking defense under MGO-induced carbonyl stress.

Exploratory untargeted brain metabolomics under MGO-induced carbonyl stress showed that LFEAN031 partially modulated global brain metabolome dysregulation, as supported by PERMANOVA (F = 3.51, R^2^ = 0.46, *p* = 0.026) (Figure S6A, S6B and Data S7). Notably, three lysophospholipids, such as PG (44:12), PG (34:1) and PS (16:0/18:1), together with alanine and 2-hydroxyglutaric acid, showed MGO-induced decreases and LFEAN031-associated recovery-like increases in brain metabolome (Figure S6D). However, unlike the cecal metabolome, the predefined MGO-AGEs breaking candidate did not show a coherent recovery pattern, suggesting that the observed brain effects of LFEAN031 may involve changes in membrane lipids and redox-linked metabolites (Figure S6C). For example, Trp and Asn showed further decreases with LFEAN031 under MGO, and the three GGPs were not detected in brain. Thus, although LFEAN031 partially modulated MGO-induced brain metabolome dysregulation, we did not find direct evidence that the intestinal MGO-AGEs breaking candidate panel, defined by the GGP pool and the aromatic axis, is preserved in the brain.

### LFEAN031 postbiotic extract coordinately modulates GGP-thiol-redox metabolism and tryptophan catabolism under carbonyl stress

To further explore the metabolomic changes associated with the identified MGO-AGEsbreaking candidates, we extended our analysis beyond the initially observed GGP pool and Trp-related aromatic metabolites. Specifically, we expanded our analysis to the *γ*-glutamyl cycle together with taurine and thiol-related redox markers, and to the three major Trp pathways: kynurenine, serotonin, and indole. We mapped the relative abundances of pathway-associated metabolites across groups and their phenotypic correlations.

**Figure 6.**
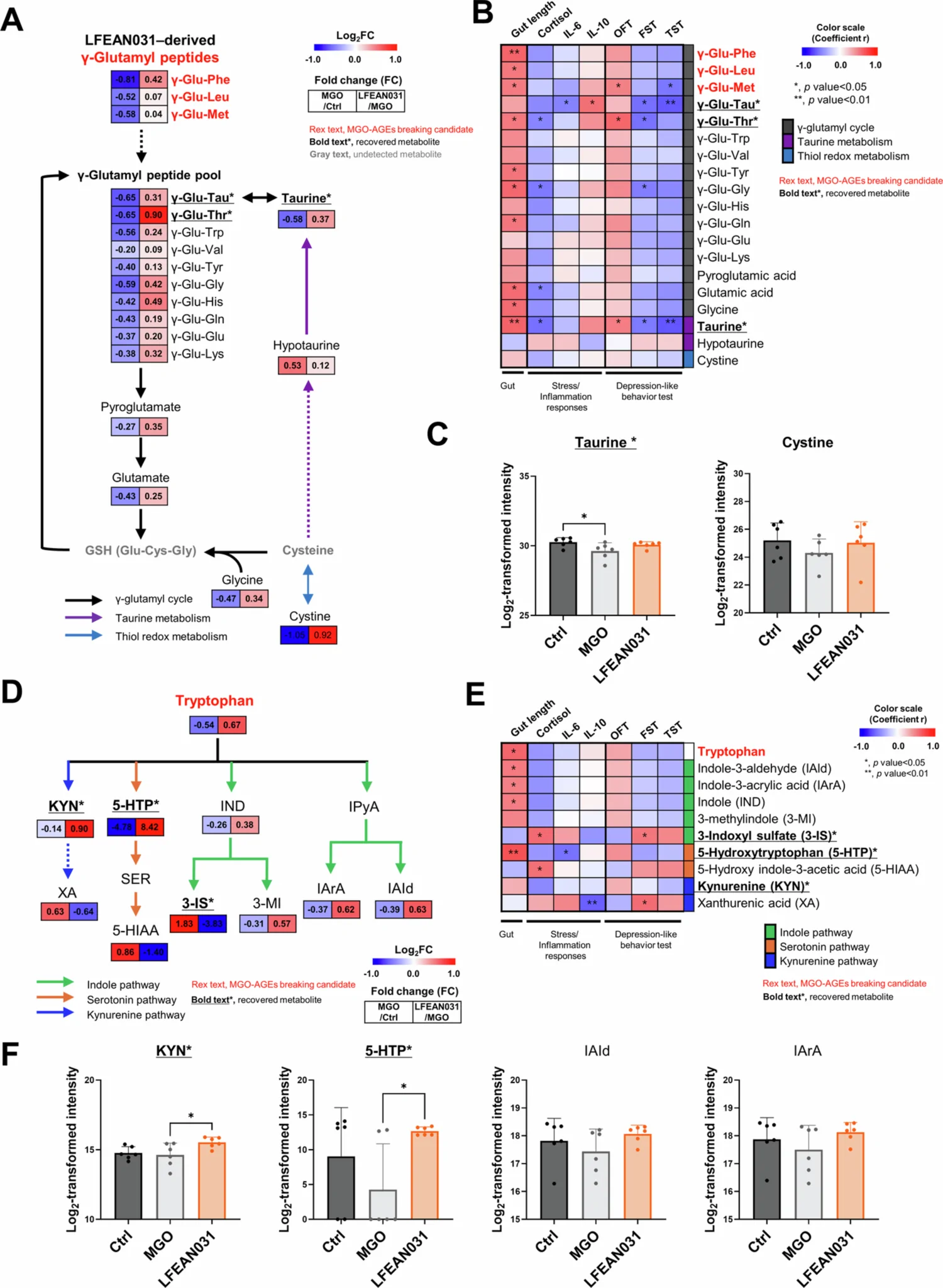
Postbiotic extract LFEAN031 modulated GGP-redox and tryptophan pathways linked to gut-brain related phenotypes. (A, D) Schematic representations of *γ*-glutamyl peptide (GGP)-redox metabolism and tryptophan (Trp) catabolism. Pathway-related metabolites in GGP-related metabolism were extended to include the *γ*-glutamyl cycle, taurine metabolism and thiol-redox metabolism (A). Trp catabolism included indole (IND), kynurenine, and serotonin branches (D). The heatmaps represent relative metabolic changes in the MGO vs Ctrl and LFEAN031 vs MGO comparisons. (B, E) Heatmaps showing correlations between relative abundances of GGP-related (B) and Trp-related metabolites (E) following LFEAN031 treatment under carbonyl stress, and observed phenotypes associated with gut–brain dysfunction. Data are presented as means ± standard error of the mean (mean ± SEM, *n* = 6). (C, F) Box plots showing selected metabolites involve in GGP-related metabolism (C) and Trp-related metabolism. Taurine and cystine were shown in (C), whereas kynurenine (KYN), 5-hydroxytryptophan (5-HTP), indole-3-aldehyde (IAld), and indole-3-acrylic acid (IArA) are shown in (F). Metabolite names shown in bold indicate metabolites that differed significantly between MGO group and either the control or LFEAN031-treated group. Metabolite names shown in red indicate metabolites selected as MGO-AGEs breaking candidates. Asterisks indicate statistically significant differences compared with the MGO group (*p < 0.05, **p < 0.01, ***p < 0.001; one-way ANOVA followed by Tukey’s *post hoc* test).

In the extended analysis of the GGP pool, we observed a coherent, recovery-like pattern in which MGO exposure decreased metabolite abundance and LFEAN031 treatment increased it. All 13 detected GGPs, including the previously identified MGO-AGEs breaking candidates and the two recovered GGPs, followed this pattern (Figure 6A, Data S8). Within the investigated pathways, pyroglutamate, glutamate, taurine, and cystine showed a consistent directional pattern, paralleling this recovery-like trend (Figure 6A, C). Correlation analysis showed that several GGPs, including three MGO-AGEs breaking candidates, glutamate, and pyroglutamate showed significant positive correlations with colon length, consistent with their recovery-like increase following LFEAN031 treatment. Taurine and the two recovered GGPs generally displayed significant correlations with stress and depression-like behavior readouts (serum cortisol, FST, and TST), being associated with lower stress markers or reduced immobility times (Figures 6B, S5A). These results indicated a coherent, recovery-like modulation of the GGP pool, the *γ*-glutamyl cycle, and thiol-redox metabolism (taurine, cystine) under carbonyl stress, which may be associated with gut-brain axis function.

LFEAN031 treatment was associated with bidirectional changes across three Trp pathways under MGO-induced carbonyl stress (Figures 6D, S5B, Data S8). MGO exposure decreased Trp levels while increasing the uremic co-metabolite 3-IS. Compared withthe MGO group, LFEAN031 treatment significantly decreased 3-IS levels and increased those of kynurenine (KYN) and the serotonin precursor 5-hydroxytryptophan (5-HTP). Several neuroprotective and cytoprotective compounds, including indole (IND), indole-3-aldehyde (IAld), and indole-3-acrylic acid (IArA), showed a more moderate increasing trend (Figure 6F). Correlation analysis revealed that 3-IS showed a significant positive correlation with the levels of serum cortisol and immobility times in the FST, whereas 5-HTP and indole derivatives exhibited significant positive correlations with colon length and negative correlations with the inflammation marker IL-6 (Figures 6E, S5C–D). These data indicated that LFEAN031 treatment was associated with compound-specific shifts across the kynurenine, serotonin, and indole branches of Trp catabolism, pointing to a possible link to gut-brain axis function.

Taken together, these extended metabolomic analyses reinforce that the protective effects of LFEAN031 extend beyond the initially prioritized marker compounds, encompassing coordinated changes across the GGP-redox and Trp metabolic networks under carbonyl stress.

### *γ*-Glu-Met, a prioritized GGP marker, exhibits bioactivity in a dexamethasone-induced atrophy model

Within this metabolite-guided framework, we identified Trp, Asn, and three GGPs as enriched metabolites of the high-activity chemotype and confirmed the *in vivo* efficacy of the Trp-, Asn-, GGPs-enriched postbiotic extract LFEAN031 in an MGO-induced gut-brain dysfunction model. However, whether these individual candidate metabolites can exert direct bioactivity on their own, independent of the whole extract, had not been examined. *γ*-Glu-Met, whose biological activities remain poorly characterized, was therefore selected for individual *in vivo* evaluation as a representative GGP marker of the high-activity chemotype. In addition, we previously reported that the source strain of LFEAN031, *L. fermentum* KCTC15072BP, confers protective effects against DEX-induced muscle atrophy in both live-cell and heat-killed-cell forms.^36^ Building on these metabolite-guided and strain-level insights, we administered *γ*-Glu-Met alone in a DEX-induced muscle atrophy model to evaluate whether this individual metabolite exerts direct *in vivo* physiological activity (Figures S7A, S8A–C).

Fourteen days of continuous DEX administration induced severe skeletal muscle wasting, characterized by a significant loss in hindlimb muscle mass, decreased myofiber cross-sectional area (CSA), and compromised physical performance. Remarkably, oral administration of *γ*-Glu-Met alone (5 or 20 mg/kg/day) effectively protected against these systemic and functional impairments. *γ*-Glu-Met treatment preserved the wet weight of major hindlimb muscles, including the gastrocnemius (GCM), quadriceps femoris (QD), and plantaris (PLA) (Figures S7B, S7C, S7E, S8D–F). Consistent with these findings, Sirius red staining revealed reduced collagen deposition in the QD and GCM muscles following *γ*-Glu-Met treatment, indicating attenuated DEX-induced muscle fibrosis (Figure S9B). These structural improvements translated into significant functional recovery, restoring forelimb grip strength and treadmill exercise endurance to levels comparable to the positive control, oxymetholone (OXY) (Figures S7D, S7F, and S9A).

At the molecular level, Western blots analysis of GCM muscle tissue revealed that DEX exposure induced a highly catabolic state by upregulating the muscle-specific E3 ubiquitin ligases Atrogin-1 and MuRF1; however, co-treatment with *γ*-Glu-Met markedly suppressed these degradation markers, maintaining cellular proteostasis (Figure S7G). Furthermore, *γ*-Glu-Met administration promoted myogenic signaling by restoring the protein expression of myogenic transcription factors (MyoD and Myogenin) as well as the structural protein MyHC (Figure S9C, S9D).

These results demonstrate that *γ*-Glu-Met exerts direct physiological activity, extending beyond its role as a chemotype marker. More broadly, they highlight the value of strain-resolved metabolomics both for identifying functionally active postbiotic extracts and for prioritizing individual metabolites for further functional validation.

## Discussion

In this study, we established a strain-resolved metabolomic framework to enable the rational discovery of functional postbiotic candidates associated with MGO-AGEs breaking activity. Using this approach, we identified *L. fermentum* KCTC 15072BP as an active metabolite-enriched strain and demonstrated the *in vivo* efficacy of its postbiotic extract, the intracellular metabolite extract LFEAN031, in a mouse model of MGO-induced carbonyl stress. LFEAN031 mitigated MGO-induced gut barrier disruption, HPA axis hyperactivation, and depressive-like and anxiety-like behavior. Cecal metabolomic profiling revealed that postbiotic extract treatment was associated with modulation of Trp catabolism and GGP-linked redox metabolism that had been altered under carbonyl stress.

Our framework provides a strategy complementary to conventional strain selection approaches used in probiotic and postbiotic research. Traditionally, the selection of active strains has relied primarily on phenotypic evaluation, with subsequent metabolic profiling conducted to identify active compounds or elucidate their mechanisms of action.^18^ By contrast, our framework employs metabolomic-based chemotaxonomy as a discovery engine to identify functional chemotypes and bioactive metabolites prior to biological validation. In addition, unlike many previous postbiotic studies that mainly analyzed cell-free supernatants (CFS) or lysates, we focused on microbial intracellular metabolite extracts. This strategy enables the detection of relatively high concentrations of bioactive and signaling metabolites while improving analytical stability and reproducibility, thereby complementing the limited chemical specificity often observed in CFS-based analyses.^37^ ^,^ ^38^

Using this approach, species-level classification was insufficient to predict MGO-AGEs breaking activity, whereas chemotype analysis successfully captured functional heterogeneity among strains of *L. fermentum*. These findings support the accumulating evidence that probiotic functionality is not necessarily a universal species-level trait but can instead arise from strain-specific metabolic characteristics, particularly those associated with intracellular metabolite profiles.^19^ ^,^ ^20^ Our observations are consistent with those of previous chemotaxonomic studies on *L. plantarum*, where distinct metabolic chemotypes were associated with clear differences in antioxidant activity, suggesting that functional chemotype analysis may represent a broadly applicable strategy for linking microbial metabolism to functional bioactivity.^23^ Taken together, these results highlight the importance of strain-level metabolic signatures in guiding the rational identification of functional microbial candidates.

Through this strain-level metabolomics framework, we identified marker compounds, including Trp, Asn, and three GGPs, representing the metabolic signatures of the high-antiglycation chemotype. Subsequent *in vitro* assays demonstrated that these metabolites possess potent MGO-AGEs breaking activities. While Asn and the three prioritized GGPs exhibited linear, dose-dependent increases in their protective profiles, Trp displayed a distinct biphasic curve, reaching peak efficacy followed by a progressive decline at higher, supraphysiological concentrations (Figure 3F). One plausible explanation for this concentration-dependent decline in activity is that the balance between carbonyl-scavenging and pro-oxidant activities of Trp shifts in a concentration-dependent manner. At moderate concentrations (*e.g*., 50 µM), the electron-rich indole ring of Trp may act as an effective nucleophilic scavenger of reactive dicarbonyl species. However, at higher concentrations, Trp may exhibit pro-oxidant behavior, potentially through concentration-dependent autooxidation and generation of reactive oxygen species (ROS), thereby offsetting its antiglycation activity. Despite this non-linear *in vitro* profile, Trp availability has been shown to causally regulate depressive-like behavior in a Trp-depletion model, and, more directly relevant to carbonyl stress, our previous study demonstrated that Trp supplementation ameliorated MGO-induced depressive-like behavior, memory impairment, and oxidative stress *in vivo*, supporting its protective relevance under carbonyl stress conditions.^27^ ^,^ ^39^

The this functional profile further invites comparison with established synthetic and endogenous anti-glycation agents, such as Alagebrium (ALT-711) and cysteine.^40^ Alagebrium, a well-characterized synthetic AGEs breaker, selectively targets and cleaves established-carbonyl-derived cross-links within consolidated AGEs complexes. The electron-rich indole ring of Trp may serve as a nucleophile under physiological conditions. This functional motif may interact with highly electrophilic carbonyl carbons involved in MGO-mediated protein-protein cross-linking, potentially contributing to non-enzymatic breakdown of accumulated glycation adducts. Furthermore, the protective roles of our identified GGPs may share functional similarities with the well-established anti-glycation properties of cysteine. Cysteine is known to directly trap reactive dicarbonyl intermediates *via* its nucleophilic thiol and amino groups, or reversibly modulate Amadori transformation structures. Similarly, the structural architecture of the prioritized GGPs may enable direct, non-enzymatic interaction with reactive carbonyl species. These peptides may serve as intermediates or metabolic indicators of the *γ*-glutamyl cycle linked to GSH homeostasis, suggesting an indirect connection to GSH-dependent dicarbonyl-detoxification.^41^ ^,^ ^42^ Taken together, these pathways suggest that Trp and GGPs may act similarly to known AGEs-breaking agents, thereby reducing glycation burden under carbonyl stress. Previous studies further showed that MGO-derived AGEs breakers may improve vascular protein flexibility in aged models.^43^ ^,^ ^44^

Guided by the strain selection strategy based on the enrichment of MGO-AGEs breaking marker metabolites, the intracellular metabolite extract (LFEAN031) derived from *L. fermentum* KCTC15072BP demonstrated protective effects against MGO-induced intestinal and behavioral dysfunction. MGO exposure has been shown to induce gut dysbiosis and disrupt intestinal barrier integrity, which may facilitate the translocation of bacterial metabolites into the systemic circulation.^32^ ^,^ ^45^ The resulting leaky gut can exacerbate neurotoxic processes by activating the HPA axis and elevating pro-inflammatory cytokine levels, thereby contributing to the development of behavioral abnormalities.^46^ ^,^ ^47^ In this study, LFEAN031 administration significantly mitigated these pathological alterations, including the improvement of intestinal integrity, inflammatory markers, and HPA axis signaling. These results suggest that postbiotic-derived metabolites are associated with maintaining intestinal homeostasis and stress responses under carbonyl stress conditions. Consistent with these physiological improvements, behavioral assessments revealed diminished immobility and enhanced exploratory activity, indicating attenuation of depressive- and anxiety-like behaviors. Collectively, these findings indicate that active metabolite-enriched postbiotic extract, LFEAN031, mitigates intestinal barrier disruption, systemic stress, inflammatory responses, and depressive- and anxiety-like behaviors associated with MGO-induced carbonyls stress.

The protective effects of the postbiotic extract were accompanied by modulation of intestinal metabolic alterations induced by MGO exposure. Untargeted metabolomic profiling indicated that MGO-induced stress shifts the gut metabolome, with relative accumulation of undigested carbohydrates such as maltotriose and stachyose and concurrent decrease in several amino acids and lipid-related metabolites. MGO has been reported to damage microbial components and to impair intestinal barrier integrity, thereby increasing luminal oxygen tension and destabilizing obligate anaerobic communities^.12,13^ In particular, MGO exposure has been shown to selectively deplete acetate/butyrate-producing genera, including *Harryflintia*, *Intestinimonas* and members of Ruminococcaceae, in association with reduced SCFA concentrations.^13^ ^,^ ^48^ A similar disruption may plausibly underlie the accumulation of undigested carbohydrates observed in our study, as these substrates are normally fermented by anaerobic microbes for SCFA production.^49^ In addition, the decreased levels of several amino acids, fatty acids, and nucleotide-related metabolites showed a recovery-like pattern following postbiotic treatment, consistent with the attenuated mucosal damage and oxidative depletion under MGO-induced stress.^50^ ^,^ ^51^

Beyond this general metabolic modulation, we further examined whether the antiglycation-associated metabolites enriched in LFEAN031 reflected broader pathway-level alterations and correlated with the observed phenotypic outcomes. Cecal levels of three *γ*-glutamyl marker compounds, *γ*-Glu-Met, *γ*-Glu-Leu, *γ*-Glu-Phe, were reduced following MGO exposure and subsequently increased following LFEAN031 administration.^52^ ^,^ ^53^ In addition, multiple GGPs showed a pattern of MGO-induced decrease followed by modulation under postbiotic treatment, consistent with coordinated changes in pyroglutamate, glutamate, taurine and cystine across the *γ*-glutamyl cycle, taurine metabolism, and thiol-redox markers. Notably, the recovery of GGPs and taurine was associated with improvements in stress- and behavior-related outcomes, including reduced cortisol levels and immobility time. MGO-induced carbonyl stress has been reported to impair thiol-redox systems, including glutathione (GSH) and cysteine/cystine pool, mechanistically linked to *γ*-glutamyl cycle regulation. In the brain, such disturbances have been associated with neuroinflammatory responses and depressive-like behaviors.^54-57^ Taken together, these findings suggest that postbiotic-driven modulation of *γ*-glutamyl cycle, taurine metabolism, and thiol-redox pathways is associated with the observed improvements in stress- responses as well as depression- and anxiety-like behavioral under carbonyl stress.

In parallel with these redox-related changes, Trp catabolism emerged as a second major axis of MGO-induced metabolic dysregulation, which was partially normalized by postbiotic treatment. MGO exposure has been reported to glycate and deplete the Trp pool, thereby inducing a metabolic deficit associated with suppression of the serotonin pathway, alteration of IND signaling, and acceleration of the kynurenine pathway.^27^ ^,^ ^41^ In accord with this concept, postbiotic administration appeared to partially redirect Trp metabolism toward pathways associated with neurophysiological homeostasis in the intestine. Notably, the levels of 3-IS, a host–microbe co-metabolite linked to systemic inflammation and neurotoxicity, were significantly reduced by postbiotic treatment and positively correlated with serum cortisol levels and depressive-like behaviors.^35^ ^,^ ^58^ We also observed decreased levels of the neurotoxic kynurenine metabolite XA, indicating a modulation of aromatic amino acid metabolism toward a less neurotoxic profile.^34^ ^,^ ^35^ In contrast, the abundance of protective IND derivatives (IAld and IArA) and the serotonin precursor 5-HTP increased following postbiotic administration. These metabolites have been reported to contribute to intestinal barrier protection and modulation of the enteric nervous system (ENS) function.^59^ ^,^ ^60^ Although compositional microbiome data were not obtained in the present study, these Trp-related metabolic shifts may be coupled with underlying changes in gut microbial community structure. Supporting this possibility, other MGO exposure models have shown that dietary interventions improve indole-derived metabolites and behavioral outcomes, together with shifts in specific gut taxa, notably a reduction in pro-inflammatory *Helicobacter hepaticus*.^61^

Collectively, these cecal metabolomic findings indicate that postbiotic extract administration was associated with a coordinated modulation of multiple metabolic axes disrupted by MGO-induced carbonyl stress, encompassing the *γ*-glutamyl cycle and thiol-redox metabolism as well as Trp catabolism. However, within these proposed pathways, several key downstream metabolites, such as serotonin, GSH, and cysteine, were not detected or fell below the detection threshold in the present dataset, and targeted quantitative validation of these specific metabolites will be required to substantiate the mechanistic links proposed here.

Beyond the cecal metabolome, exploratory brain metabolomic profiling (*n* = 3-4) indicated that LFEAN031 partially modulated the MGO-induced dysregulation in brain (PERMANOVA, *p* = 0.026), suggesting potential gut-brain axis engagement at the central level (Figure S6A). However, the metabolite signature underlying this brain-level modulation differed from the intestinal MGO-AGEs breaking candidate panel. Under LFEAN031 treatment, Trp and Asn showed further MGO-associated decreases, and GGPs were not detected in brain tissue. Instead, several lysophospholipids and 2-hydroxyglutaric acid exhibited recovery-like patterns. MGO exposure has been reported to disrupt amino acid metabolism and membrane phospholipid composition through glycation of proteins and phospholipids, which may partly underlie the brain-level changes observed in this study. ^62-64^ Given the exploratory nature of these findings, further studies with larger cohorts and brain-targeted metabolite panels will be needed to clarify whether and how gut-derived metabolites contribute to the central metabolic alterations.

The protective effects of the prioritized *γ*-Glu–Met in the DEX-induced muscle atrophy model indicate that this marker compound possesses direct bioactivity, supporting the validity of our metabolite-guided prioritization strategy beyond the original gut-brain model (Figures S7–S9). While dicarbonyl (MGO) stress was utilized as our primary screening criterion, carbonyl stress is widely recognized as a systemic driver of cellular dysfunction affecting multiple tissues, including skeletal muscle.^27^ ^,^ ^65^ Indeed, our source strain, *L. fermentum* KCTC15072BP, was previously shown to protect against glucocorticoid-induced muscle wasting.^36^ Although GGPs are metabolically linked to the *γ*-glutamyl cycle and GSH-related redox metabolism, their direct bioactive roles in physiological conditions such as muscle atrophy have remained poorly characterized.^52^ ^,^ ^53^ By demonstrating that a single prioritized GGP marker, *γ*-Glu-Met, can independently reproduce this muscle-protective phenotype, we support the notion that our functional chemotyping strategy successfully prioritizes bioactive molecules rather than mere chemotype biomarkers.

Although the MGO and DEX models represent distinct pathological conditions, they share several stress response mechanisms, including carbonyl stress, oxidative stress, mitochondrial dysfunction, and impaired neuromuscular proteostasis.^52^ ^,^ ^66^ Rectal MGO administration induces severe hypothalamic-pituitary-adrenal (HPA) axis hyperactivation and cortisol release, exposing the host to endogenous glucocorticoid stress as a state paralleled by exogenous DEX administration. At the subcellular level, both dicarbonyl and glucocorticoid exposure converge upon the generation of excessive oxidative stress and mitochondrial dysfunction, which ultimately compromises cellular proteostasis. While this proteostasis collapse impairs tight junction assembly in the gut, it upregulates muscle-specific E3 ubiquitin ligases (Atrogin-1 and MuRF-1) in skeletal muscle, driving muscle wasting. Thus, the muscle-protective effects of *γ*-Glu-Met observed in the DEX-induced atrophy model may be linked to its proposed roles as an MGO-AGE breaker and contributor to GSH homeostasis, potentially involving shared stress-response pathways.

Despite the significant findings of this study, it had certain limitations. First, although a cohort size of *n* = 6 per group is widely accepted in preclinical rodent studies, the relatively small sample size utilized for behavioral and tissue-level analyses may limit the generalizability and statistical resolution of our physiological findings. Second, although postbiotic extract administration altered intestinal and brain metabolic profiles, the absence of microbiome compositional data limits our ability to define causal links between metabolic alterations and CNS responses. Future studies incorporating 16S rRNA or shotgun metagenomic sequencing, will be required to delineate the roles of specific taxa and pathways in the observed gut-brain phenotypes. Third, although the postbiotic extract used in this study contained multiple metabolites, including the identified MGO-AGEs breaking metabolites, the comprehensive identification of active compounds and their relative contributions remain unclear. As postbiotics generally exert their effects through complex mixtures rather than *via* single molecules, further studies are warranted to dissect the synergistic interactions among these metabolites. Finally, although our metabolomic platform demonstrated efficacy in current models, its generalizability and robustness across diverse disease contexts require further validation.

## Conclusion

**Figure 7.**
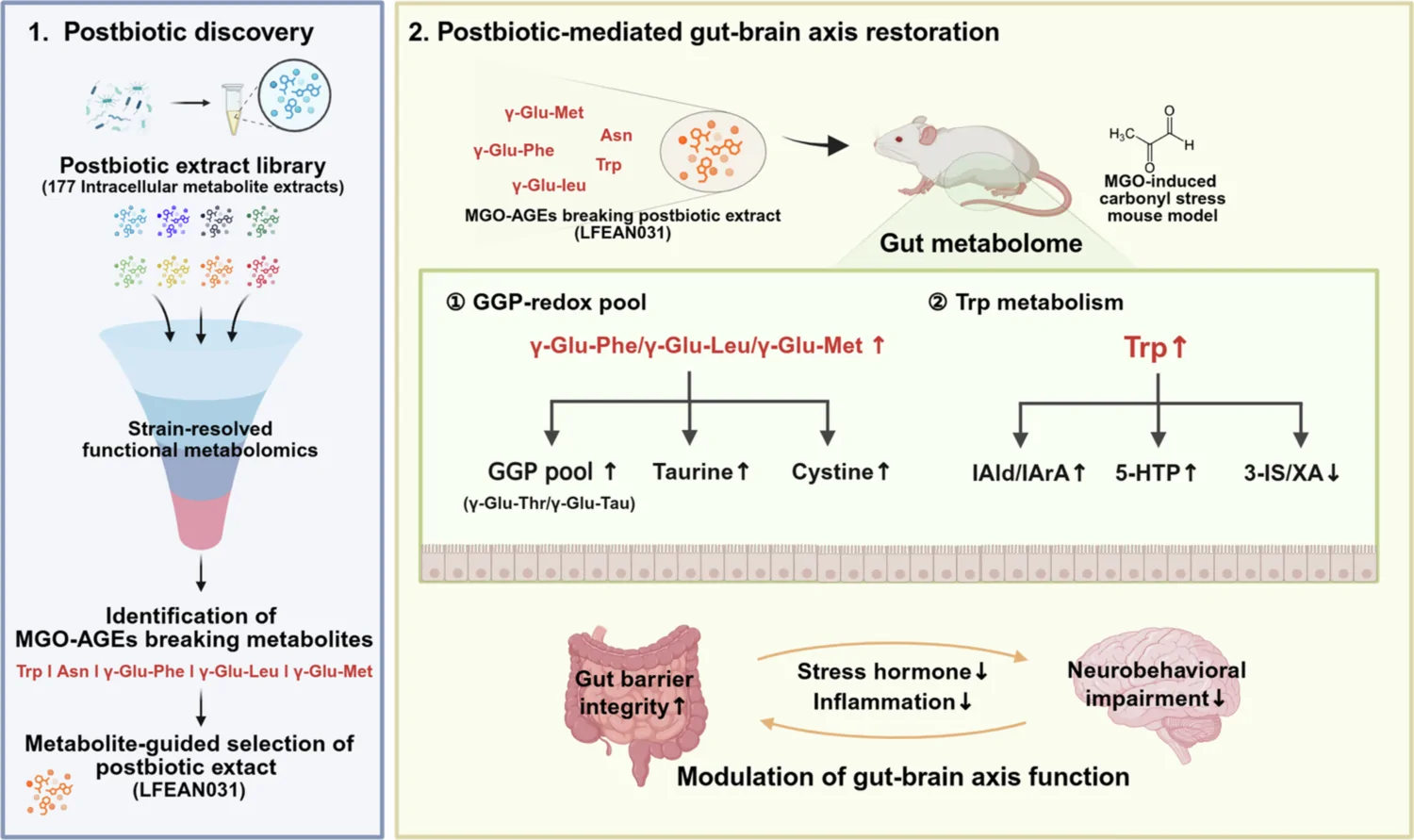
Proposed model of postbiotic extract LFEAN031-associated modulation of the gut-brain axis *via* coordinated GGP-redox and tryptophan metabolism. A metabolite-guided discovery framework identified a postbiotic extract, LFEAN031, derived from *Limosilactobacillus fermentum*, characterized by enrichment of MGO-AGEs breaking activity compounds, including tryptophan (Trp), asparagine (Asn), and three *γ*-glutamyl-peptides (GGPs). LFEAN031 was orally administered in a methylglyoxal (MGO)-induced carbonyl stress model. Cecal metabolome analysis revealed two coordinated metabolic arms. In the GGP-redox pool, LFEAN031 treatment increased several GGPs, including the MGO-AGE breaking metabolites and extended members (*γ*-Glu-Tau and *γ*-Glu-Thr), together with redox-related metabolites such as taurine and cystine, all showing recovery-like patterns under carbonyl stress. In parallel, increased Trp was accompanied by higher levels of protective indole derivatives [indole-3-aldehyde (IAld) and indole-3-acrylic acid (IArA)] and the serotonin precursor 5-hydroxytryptophan (5-HTP), alongside reduced levels of neurotoxic metabolites 3-indoxyl sulfate (3-IS) and xanthurenic acid (XA). These gut metabolome changes were associated with improved gut barrier integrity, reduced stress and inflammatory responses, and attenuated depressive-like and anxiety-like behaviors, reflecting modulation of gut-brain axis function under carbonyl stress. Figure 7 was created using BioRender (https://BioRender.com/bkz3r1i). *γ*-Glu–Met, *γ*-Glutamyl–methionine; *γ*-Glu–Leu, *γ*-Glutamyl–leucine; *γ*-Glu–Phe, *γ*-Glutamyl–phenylalanine. *γ*-Glu–Tau, *γ*-Glutamyl–Taurine; *γ*-Glu–Thr, *γ*-Glutamyl–Threonine.

In conclusion, this study demonstrates that strain-resolved activity metabolomics provides an effective framework for discovering functional postbiotic candidates based on metabolite signatures. Through metabolomic-guided strain selection, we identified and validated a postbiotic extract (LFEAN031) that alleviates carbonyl stress-associated intestinal disruption and depressive- and anxiety-like behaviors. LFEAN031 extract administration was associated with modulation of intestinal metabolic signatures, including bidirectional changes of Trp catabolism, and GGP-redox pool (Figure 7). Beyond the gut-brain axis findings, *γ*-Glu-Met exerted direct physiological effects in an independent DEX-induced muscle atrophy model, transcending its original role as a chemotype marker and supporting the value of this framework for prioritizing individual metabolites for further functional validation. Together, our findings provide a proof-of-concept for a metabolite-guided strategy for postbiotic discovery and highlight the potential of microbiota-derived metabolites as modulators of gut–brain axis function under carbonyl stress.

## Supplementary Material

263778949_Re_Revised_Supplementary_material_Clean_260804.docx

263778949_Supplementary_Data.xlsx 263778949_Supplementary_Data.xlsx

## Data Availability

The datasets generated from the postbiotic library screening and cecal metabolome in this study have been deposited in the MassIVE public repository with accession numbers MSV000099214, MSV000099215, and MSV000101307. All the data supporting the findings of this study are included in the Supplementary Materials.
