# Supplementary material for: Strain-resolved metabolomic chemotyping identifies tryptophan and γ-glutamyl peptides as MGO-AGEs breaking postbiotic compounds for carbonyl stress mitigation
Source: Gut Microbes. 2026 Sep 12;18(1):2728833. doi: 10.1080/19490976.2026.2728833 (PMC13577272; doi:10.1080/19490976.2026.2728833)
Supplement: Supplementary file 1 [file KGMI_A_2728833_SM6049.docx]

**Supplemental Materials**

**Strain-resolved metabolomic chemotyping identifies tryptophan and γ-glutamyl peptides as MGO-AGEs breaking postbiotic compounds for carbonyl stress mitigation**

Su-Hyun Kim, Seong-Min Hong, Eun Yoo Lee, Seul-Ah Kim, Nam Soo Han, Sun Yeou Kim and Choong Hwan Lee

**Contents**

- Supplemental Methods
- Supplemental Figures (Figures S1―S9)
- Supplemental Tables (Tables S1―S2)
- Supplemental Data Legends

**Additional supplemental file:**

- Supplemental Data (Data S1―S8; Microsoft Excel workbook)

# **Supplemental Methods**

## **Evaluation of a representative *γ*-glutamyl peptide in a DEX-induced muscle atrophy model**

### Animals and experimental design

For the dexamethasone (DEX)-induced muscle atrophy model, eight-week-old male C57BL/6N mice (Orient Bio, Seongnam, Korea) were housed under controlled environmental conditions (23 ± 1°C and 60 ± 5% humidity, 12 h light/dark cycle) with *ad libitum* access to standard chow and water. After a one-week acclimatization period, mice were randomly assigned to five groups (n = 8 per group): Control (Ctrl, vehicle-treated), DEX, DEX + 50 mg/kg/day of oxymetholone (OXY, *p.o.*), DEX + 5 mg/kg/day of γ-Glu-Met (GM 5, *p.o.*), and DEX + 20 mg/kg/day of γ-Glu-Met (GM 20, *p.o.*) (See Figure 6A). DEX was administered subcutaneously at 20 mg/kg/day for 14 consecutive days, while γ-Glu-Met or OXY was administered orally once daily. Mice exhibiting clinical abnormalities or ≥20% weight loss were humanely euthanized using CO_2_ (30–70% volume/min; AVMA guidelines) with death confirmed after ≥5 min of respiratory and cardiac arrest, under approval by the Institutional Animal Care and Use Committee of Gachon University (Incheon, Korea; Approval No. GU1-2024-IA0046).

### Treadmill endurance and grip strength tests

To evaluate physical performance, treadmill endurance and grip strength tests were conducted at baseline and on days 7 and 14. Forelimb grip strength was measured using a grip strength meter (BIO-G53, BIOSEB, Vitrolles, France). Each mouse was placed on a horizontal metal grid and gently pulled backward by its tail until it released its grip. The peak tension (g) was recorded three times per mouse, and the average value was used for analysis. Treadmill endurance was assessed using a motorized treadmill (JD-A-22, Jungdo B&P, Seoul, Korea). Following a one-week adaptation period, the mice performed a 30-minute running protocol: a 5-minute warm-up at 5 m/min, followed by 20 minutes of acceleration (5-20 m/min), and a 5-minute cool-down at 10 m/min. Total running time (s) and average speed (m/min) were recorded^31^.

### Muscle mass

At the time of sacrifice, skeletal muscles including quadriceps femoris (QD), gastrocnemius (GCM), soleus (SOL), plantaris (PLA), and extensor digitorum longus (EDL) were carefully dissected, and their wet weights were recorded. Relative muscle mass was calculated by normalizing each muscle’s weight to the final body weight (mg/g).

### Histology and Sirius red staining

QD and GCM muscle tissues were fixed in 10% neutral-buffered formalin, paraffin-embedded, and sectioned at a thickness of 4 μm. Sections were stained with hematoxylin and eosin (H&E) to assess morphological changes and with Sirius red to evaluate collagen deposition. Images were captured using a light microscope (Olympus BX53, Tokyo, Japan) at 20×–40× magnification. The cross-sectional area (CSA) was determined by measuring at least 30–50 representative myofibers per section across multiple non-overlapping fields of view using ImageJ software).

### Micro-CT analysis

Calf muscle morphology was evaluated using a high-resolution micro-CT scanner (SkyScan 1176, Bruker, Kontich, Belgium). Fixed hind limbs were scanned using the following parameters: 50 kV voltage, 200 μA current, 0.5 mm aluminum filter, 9 μm voxel resolution, and 0.4° rotation steps. Image reconstruction was performed using NRecon software (Bruker). The CSA of the calf and the tibia–fibula length were measured at the mid-calf level using the manufacturer’s CTAn software for quantitative analysis.

### Western blot analysis of muscle tissue

GCM muscle tissues were homogenized in RIPA buffer supplemented with protease and phosphatase inhibitor cocktails (Thermo Fisher Scientific, Waltham, MA, USA). Equal amounts of total protein were resolved by SDS-PAGE and transferred onto nitrocellulose membranes (Bio-Rad, Hercules, CA, USA). Membranes were blocked with 5% nonfat dry milk in TBST for 1 h at room temperature and subsequently incubated overnight at 4 °C with primary antibodies (1:1000 dilution) against muscle atrophy markers, including MuRF1 (Abcam, Cambridge, UK; ab172479), Atrogin-1 (Abcam; ab168372), Myogenin (Abcam; ab128544), MyoD (myoblast determination protein; Santa Cruz Biotechnology, Santa Cruz, CA, USA; sc-377460), and myosin heavy chain (MyHC; Santa Cruz Biotechnology; sc-32732). Following washing, membranes were incubated with HRP-conjugated secondary antibodies (1:2000 dilution) for 2 h at room temperature. GAPDH (Cell Signaling Technology, Danvers, MA, USA; #2118) or $\alpha$-tubulin (Sigma-Aldrich, St. Louis, MO, USA; T5168) was used as an internal loading control to ensure equal protein loading.

# **Supplemental Figures**

**Figure S1. Schematic workflow of the strain-resolved functional metabolomics framework for rational selection of a postbiotic extract candidate.**

A postbiotic extract library was constructed from 113 bacterial strains spanning three families and 10 species (Lactobacillaceae, 4 species/38 strains; Enterococcaceae, 2 species/26 strains; Bifidobacteriaceae, 4 species/49 strains). Each strain was cultured in modified MRS medium for 24 h under aerobic or anaerobic conditions, and cell pellets were collected per strain-condition combination. 177 intracellular metabolite extracts were obtained and collectively defined as the postbiotic extract library. Each extract was analyzed in untargeted metabolomics-based chemotyping and *in vitro* MGO-AGEs breaking activity assay. *In vitro* MGO-AGEs breaking activities were compared among chemotype groups at two levels of resolution: species-level chemotypes (PGCA-PGCE) and, within the single *L. fermentum*/anaerobic postbiotic group (LFEAN), strain-resolved chemotype groups (LFCA-LFCC). The strain-level analysis provided higher functional resolution and identified LFCB as the strain-level chemotype with the highest MGO-AGEs breaking activity. Five marker compounds, including tryptophan (Trp), asparagine (Asn), *γ*-Glu–Phe, *γ*-Glu–Leu, and *γ*-Glu–Met, were identified based on LFCB-specific enrichment and their functional association with this activity. Marker compounds-guided selection prioritized LFEAN031 as the representative postbiotic extract, which was validated *in vivo* in an MGO-induced carbonyl stress mouse model. *γ*-Glu–Met, *γ*-Glutamyl–methionine; *γ*-Glu–Leu, *γ*-Glutamyl–leucine; *γ*-Glu–Phe, *γ*-Glutamyl–phenylalanine. *γ*-Glu–Tau, *γ*-Glutamyl–Taurine; *γ*-Glu–Thr, *γ*-Glutamyl–Threonine.

**Figure S2. Chemical diversity and specific marker compounds of 16 postbiotic groups based on untargeted metabolic profiles**

**(A)** PCA score plot, (**B)** PLS-DA score plot, and (**C)** PLS-DA loading plot of 177 intracellular metabolite extracts from postbiotic library, analyzed using GC–MS and LC–MS. (**D)** Heatmap illustrates the relative abundance of group-specific marker compounds. Marker compounds for each postbiotic group are defined as significantly differential metabolites with a fold change > 3.0 (indicated by #, FC > 3) or 4.0 (indicated by ##, FC > 4) relative to the overall sample average. Postbiotic group definitions are provided in Table 1 and Table S1.

**Figure S3. Chemical signature and specific marker compounds in a cluster group of 10 *Limosilactobacillus fermentum* (LFEAN) strains based on untargeted metabolic profiles**

**(A)** PCA score plot of 10 LFEAN extracts analyzed using GC–MS and LC–MS. (**B)** Heatmap illustrating the relative abundance of discriminant metabolites in intracellular metabolite extracts from 10 LFEAN extracts. Cluster-specific marker compounds were defined as those with more than a 2.0-fold higher abundance compared with the average across all samples. Identified marker compounds for LFCA and LFCB are indicated in blue and red text, respectively. No marker compounds were identified in LFCC.

**Figure S4. LFEAN031 postbiotic extract modulates the global intestinal metabolome and its correlation with gut–brain axis-related phenotypes**

**(A)** Pairwise score plots of principal components (PC1–PC5) showing group-wise separation of cecal metabolic profiles. **(B)** Heatmaps showing correlations between relative abundances of 29 LFEAN031-recovered metabolites and observed phenotypes associated with gut–brain dysfunction under MGO-induced carbonyl stress. **(C, D)** Relative abundances of MGO-AGEs breaking metabolite, Asparagine **(C)** and *γ*-glutamyl peptide (GGP) cycle-associated metabolites **(D)** across groups. Data are presented as means ± standard error of the mean (mean ± SEM).

**Figure S5. LFEAN031 postbiotic extract links GGP-redox metabolism and tryptophan catabolism to gut–brain-related phenotypes**

**(A, C)** Correlation and linear regression analyses between functional phenotypes and the peak areas of metabolites associated with GGP-redox metabolism and tryptophan catabolism. Panel A shows recovered metabolites associated with GGP-redox metabolism, including *γ*-Glu-Tau, *γ*-Glu-Thr, and taurine, and Panel C shows tryptophan (Trp) and its catabolites, including indole (IND), indole-3-aldehyde (IAld), indole-3-acrylic acid (IArA), and 3-indoxyl sulfate (3-IS). Black, gray and orange dots in scatter plot indicate the Ctrl, MGO, and LFEAN031 groups, respectively. (**B**) Schematic representation of the tryptophan-related kynurenine pathway. The heatmap represents metabolic changes induced by methylglyoxal (MGO) and modulated by LFEAN031 administration.

**Figure S6. Postbiotic extract LFEAN031 modulates the global brain metabolome**

**(A, B)** Pairwise score plots of principal components (PC1–PC5) (A) and PCA (B) showing global separation of brain metabolic profiles across groups. **(C)** Relative abundances of antiglycation metabolites identified in high-potency LFEAN chemotypes following LFEAN administration. **(D)** Heatmaps showing restoration of 29 MGO-dysregulated metabolites following LFEAN031 administration.

**Figure S7.** **γ-Glu–Met attenuates dexamethasone-induced muscle atrophy and functional impairment.**

**(A)** Schematic representation of the experimental design for the dexamethasone (DEX)-induced muscle atrophy model. Eight-week-old male C57BL/6N mice were administered DEX (20 mg/kg/day, *i.p.*) with or without oral administration of **γ**-Glu–Met (5 or 20 mg/kg/day; designated as GM 5 and GM 20) for 14 consecutive days. Oxymetholone (OXY, 50 mg/kg/day, *p.o.*) served as a positive control. **(B)** Relative muscle mass of the gastrocnemius (GCM) normalized to body weight (mg/g). **(C)** Quantitative analysis of the myofiber cross-sectional area (CSA, μm^2^) in GCM tissues. Scale bar = 30–60 μm **(D)** Forelimb grip strength at weeks 1 and 2, normalized to body weight (g/g).**(E)** Representative images of hematoxylin and eosin (H&E)-stained GCM sections (Scale bar = 100 μm).**(F)** Exercise endurance measured by total running time (seconds) on a motorized treadmill at weeks 1 and 2.**(G)** Representative Western blot images and densitometric quantification of muscle-specific E3 ubiquitin ligases, MuRF1 and Atrogin-1. GAPDH was used as an internal loading control. Data are presented as means ± standard error of the mean (mean ± SEM, *n* = 3–8). Asterisks indicate statistically significant differences compared with the DEX group (**p* < 0.05, ***p* < 0.01, ****p* < 0.001; one-way ANOVA followed by *post hoc* test).

**Figure S8.** **Effects of γ-Glu–Met on body weight, food intake, and diverse hindlimb muscle mass in DEX-induced atrophy mice.**

**(A)** Trajectory of absolute body weight (g) during the 14-day experimental period across the five groups. **(B)** Percentage of body weight change relative to the initial weight at day 0. **(C)** Average daily food intake (g/day) to monitor potential anorectic effects of DEX or postbiotic treatment. (**D)** Representative micro-CT cross-sectional images of the mid-calf level and quantitative analysis of calf radius (mm) and calf thickness (mm^2^). **(E)** Representative images of H&E-stained quadriceps (QD) sections and quantification of myofiber cross-sectional area (CSA, μm^2^). Scale bar = 60 μm. **(F)** Relative muscle weights of the quadriceps (QD), plantaris (PLA), extensor digitorum longus (EDL), and soleus (SOL) normalized to the final body weight (mg/g). Data are presented as means ± standard error of the mean (mean ± SEM, *n* = 8 for body weight and muscle weights; *n* = 2–3 for Micro-CT and histological analysis). Asterisks indicate statistically significant differences compared with the DEX group (**p* < 0.05, ***p* < 0.01, ****p* < 0.001, *****p* < 0.0001; one-way ANOVA followed by *post hoc* test).

**Figure S9.** **γ-Glu–Met improves exercise capacity and promotes myogenic signaling in DEX-induced muscle atrophy.**

**(A)** Average treadmill running speed (meters/minute) measured at week 1 and week 2. γ-Glu–Met administration significantly prevented the decline in running performance induced by dexamethasone (DEX). **(B)** Representative images of Sirius red-stained sections of the quadriceps (QD) and gastrocnemius (GCM) muscles. Sirius red staining was performed to evaluate muscle morphology and collagen deposition (fibrosis). Scale bar = 60 μm. **(C)** Densitometric quantification of myogenic regulatory factors (MyoD and Myogenin) and a structural protein (MyHC) from Western blot analysis. Data are expressed as fold changes relative to the control group after normalization to GAPDH. **(D)** Representative Western blot bands showing the protein levels of Myoblast determination protein 1 (MyoD), Myogenin, and myosin heavy chain (MyHC) in muscle tissues. GAPDH was used as an internal loading control. Data are presented as means ± standard error of the mean (mean ± SEM, *n* = 8 for treadmill tests; *n* = 3 for Western blot and histological analysis). Asterisks indicate statistically significant differences compared with the DEX group (**p* < 0.05, ***p* < 0.01, ****p* < 0.001; one-way ANOVA followed by *post hoc* test).

# **Supplemental Tables**

**Table S1. Classification of species-level chemotype groups, comprising 16 postbiotic groups derived from 177 intracellular metabolite extracts.**

| No | Species-level chemotype group | Postbiotic group | Composition |  |
| --- | --- | --- | --- | --- |
|  |  |  |  |  |
| 1 | PGCA | BANAN | *Bifidobacterium animalis subsp. lactis* (Anaerobic) |  |
|  |  | BBIAN | *Bifidobacterium bifidum* (Anaerobic) |  |
|  |  | BBRAN | *Bifidobacterium breve* (Anaerobic) |  |
| 2 | PGCB | EFMAN | *Enterococcus faecium* (Anaerobic) |  |
|  |  | EFMAE | *Enterococcus faecium* (Aerobic) |  |
| 3 | PGCC | EFSAN | *Enterococcus faecalis* (Anaerobic) |  |
|  |  | EFSAE | *Enterococcus faecalis* (Aerobic) |  |
| 4 | PGCD | LPMAN | *Lactiplantibacillus plantarum* (Anaerobic) |  |
|  |  | LPMAE | *Lactiplantibacillus plantarum* (Aerobic) |  |
| 5 | PGCE | BLOAN | *Bifidobacterium longum* (Anaerobic) |  |
|  |  | LSAAN | *Ligilactobacillus salivarius* (Anaerobic) |  |
|  |  | LSAAE | *Ligilactobacillus salivarius* (Aerobic) |  |
|  |  | LRMAN | *Lacticaseibacillus rhamnosus* (Anaerobic) |  |
|  |  | LRMAE | *Lacticaseibacillus rhamnosus* (Aerobic) |  |
|  |  | LFEAN | *Limosilactobacillus fermentum* (Anaerobic) |  |
|  |  | LFEAE | *Limosilactobacillus fermentum* (Aerobic) |  |

**Table S2. Classification of strain-level chemotype groups and antiglycation activity of 10 intracellular metabolite extracts within the LFEAN postbiotic group**

| No | Species | Strain | Cultivation | Sample name | Chemotype group | Antiglycation activity (Free amine %) |
| --- | --- | --- | --- | --- | --- | --- |
| 1 | *L. fermentum* | LBF-4 | Anaerobic | LFEAN029 | LFCB | 97.09 ± 2.54 |
| 2 | *L. fermentum* | LBF-5 | Anaerobic | LFEAN030 | LFCB | 222.47 ± 9.52 |
| 3 | *L. fermentum* | LBF-6 | Anaerobic | **LFEAN031*** | **LFCB** | **229.27 ± 9.16** |
| 4 | *L. fermentum* | LBF-7 | Anaerobic | LFEAN032 | LFCC | 0.00 ± 0.00 |
| 5 | *L. fermentum* | LBF-8 | Anaerobic | LFEAN033 | LFCC | 8.27 ± 7.20 |
| 6 | *L. fermentum* | LBF-9 | Anaerobic | LFEAN034 | LFCC | 108.03 ± 2.76 |
| 7 | *L. fermentum* | LBF-10 | Anaerobic | LFEAN035 | LFCA | 0.00 ± 0.00 |
| 8 | *L. fermentum* | LBF-11 | Anaerobic | LFEAN036 | LFCA | 0.00 ± 0.00 |
| 9 | *L. fermentum* | LBF-12 | Anaerobic | LFEAN037 | LFCC | 19.83 ± 17.83 |
| 10 | *L. fermentum* | LBF-14 | Anaerobic | LFEAN038 | LFCC | 31.27 ± 27.76 |

* LFEAN031 was selected as a postbiotic candidate strain based on its high antiglycation activity and enrichment of antiglycation markers.

# **Supplementary Data Legends**

**Data S1. Information on the postbiotic library, comprising 177 intracellular metabolite extracts from 113 bacterial strains tested in this study**

**Data S2. List of discriminant metabolites across 16 postbiotic groups and LFEAN chemotype groups based on GC-TOF-MS datasets of postbiotic extracts**

**Data S3. List of discriminant metabolites across 16 postbiotic groups and LFEAN chemotype groups based on UHPLC-Orbitrap-MS datasets of postbiotic extracts**

**Data S4. MRM transitions for targeted analysis of tryptophan metabolism based on LC–triple quadrupole–MS**

**Data S5. Relative abundances of discriminant metabolites across 16 postbiotic groups and normalized peak areas for 177 individual samples.**

**Data S6. Relative abundances of discriminant metabolites across three LFEAN chemotypes and normalized peak areas for 10 individual samples.**

**Data S7. Relative abundance of observed metabolites in cecal and brain metabolomes of**

**MGO-induced mice following administration of the postbiotic extract LFEAN031**

**Data S8. Relative abundance and correlation analysis of tryptophan- and γ-glutamyl-peptide-related metabolites in cecal metabolome of MGO-induced mice following administration of the postbiotic extract LFEAN031**
